# Esters of Glucose-2-Phosphate: Occurrence and Chemistry

**DOI:** 10.3390/molecules25122829

**Published:** 2020-06-19

**Authors:** Qiang Zhang, Si-Zhe Li, Mohammed Ahmar, Laurent Soulère, Yves Queneau

**Affiliations:** Univ Lyon, Université Claude Bernard Lyon 1, CNRS, INSA Lyon, CPE Lyon, ICBMS, UMR 5246, Bât. E. Lederer, 1 rue Victor Grignard, F-69622 Villeurbanne, France; qiang.zhang@insa-lyon.fr (Q.Z.); s.li@lic.leidenuniv.nl (S.-Z.L.); mohammed.ahmar@univ-lyon1.fr (M.A.); laurent.soulere@insa-lyon.fr (L.S.)

**Keywords:** agrocinopine, glucose-2-phosphate, phosphodiesters, agrocin 84, *Agrobacterium fabrum*

## Abstract

Phosphodiesters of glucose-2-phosphate (G2P) are found only in few natural compounds such as agrocinopine D and agrocin 84. Agrocinopine D is a G2P phosphodiester produced by plants infected by *Agrobacterium fabrum* C58 and recognized by the bacterial periplasmic binding protein AccA for being transported into the bacteria before cleavage by the phosphodiesterase AccF, releasing G2P, which promotes virulence by binding the repressor protein AccR. The G2P amide agrocin 84 is a natural antibiotic produced by the non-pathogenic *Agrobacterium radiobacter* K84 strain used as a biocontrol agent by competing with *Agrobacterium fabrum* C58. G2P esters are also found in irregular glycogen structures. The rare glucopyranosyl-2-phophoryl moiety found in agrocin 84 is the key structural signature enabling its action as a natural antibiotic. Likewise, G2P and G2P esters can also dupe the *Agrobacterium* agrocinopine catabolism cascade. Such observations illustrate the importance of G2P esters on which we have recently focused our interest. After a brief review of the reported phosphorylation coupling methods and the choice of carbohydrate building blocks used in G2P chemistry, a flexible access to glucose-2-phosphate esters using the phosphoramidite route is proposed.

## 1. Introduction

**Figure 1:**
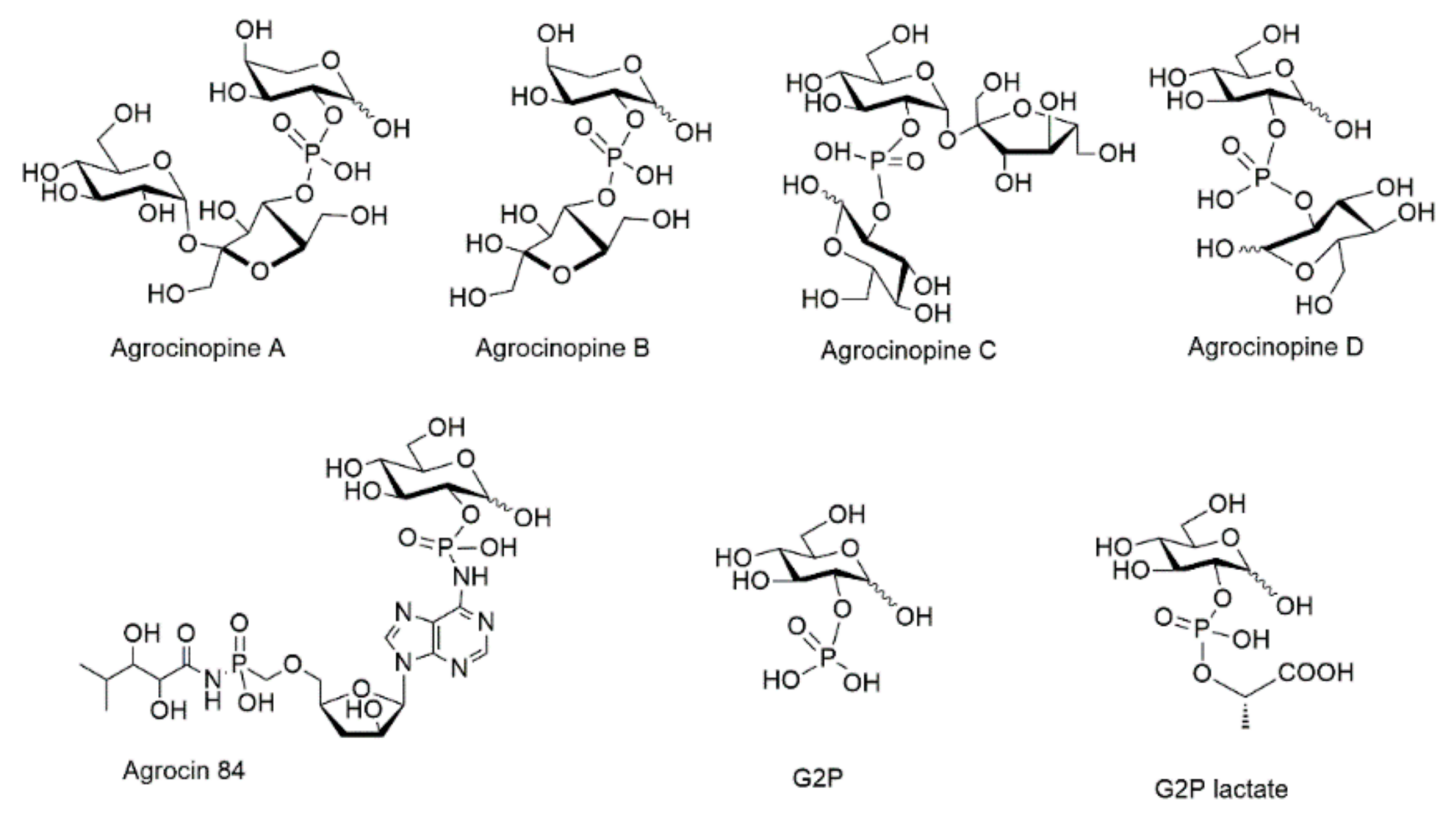
Structures of agrocinopines A, B, C and D, d-glucose-2-phosphate (G2P) and its esters G2P lactate, its amide agrocin 84.

Glucose-2-phosphate esters are rare structures, found in only a few natural compounds, among which agrocinopine D and agrocin 84. Agrocinopines are a family of opines, which are plasmid-coded enzyme products produced by pathogenic bacteria of the genus of *Agrobacterium. Agrobacterium fabrum* are Gram negative bacteria which cause tumors in plants, causing diseases such as crown gall, hairy root, and cane gall. In the process of infection by *Agrobacterium fabrum* C58*,* agrocinopines serve as nutrients and as relays in the production of quorum-sensing autoinducers. These latter subsequently control the expression of virulence genes and bacterial population [1,2]. Four agrocinopines (A, B, C and D, Figure 1) have been identified and are specific to *A. fabrum strains* C58 and *strains* Bo542 [2]. They differ in structure by being either glucose or arabinose phosphodiesters, connected with sucrose, glucose or fructose moieties (Figure 1). In *Agrobacterium fabrum*, the agrocinopine catabolism cascade involves several proteins notably AccA and AccF which are responsible for the import of agrocinopine back into the bacteria, and for the cleavage of its phosphodiester linkage, respectively.

A phosphoramidate analog of agrocinopines is the natural antibiotic agrocin 84 produced by *Agrobacterium radiobacter* K84 which is a non-pathogenic bacterial strain competing with *Agrobacterium fabrum* C58. If agrocin 84 enters into *Agrobacterium fabrum* C58 cells, its phosphodiester linkage is cleaved by AccF, releasing the toxic TM84 fragment acting as a leucine synthase inhibitor resulting in bacterial death [3]. *Agrobacterium radiobacter* can thus be used as a biocontrol agent.

The carbohydrate signature of the agrocinopines is therefore the key for the three protein-carbohydrate recognition events, first through specific recognition by the periplasmic binding proteins AccA. Then, once imported, it is recognized and hydrolyzed by the phosphodiesterase AccF. Finally, the resulting fragment binds with the repressor protein AccR responsible for virulence activation through the activation of quorum sensing signals synthesis [3].

If agrocin 84 can act as a natural antibiotic, it is because it shares the rare pyranose-2-phosphate chemical signature with agrocinopines. Agrocin 84 structure was first proposed to be a glucofuranose 1-*O*-phosphate though not ruling out other connections [4,5]. Its structure was fully identified as a glucopyranosyl-2-phosphoryl derivative after X-ray crystallography diffraction studies of its complex with AccA [6]. The ability of G2P esters to dupe the *acc* cascade of *Agrobacterium fabrum* was also confirmed using the G2P lactic acid ester (G2LP) found to be the first non-natural G2P ester to mimic the role of natural agrocinopine A [7].

Considering the importance of the G2P motif in plant pathogens, and their originality in biology compared to glucose-1-phosphates (G1P) or glucose-6-phosphates (G6P), we have further focused our attention on their chemical synthesis. Below we briefly review the chemistry of G2P and its esters, discussing the phosphorylation methods and possible carbohydrate building blocks, before applying an updated sequence to the synthesis of a short series of derivatives.

## 2. Results

Although phosphate esters are very frequent in biologically relevant carbohydrates [8], there are only a handful of G2P phosphodiesters reported in the literature. Apart from the family of agrocinopines and analogues mentioned above, the G2P motif was only found in glycogen, together with G3P and the more common G6P. Glycogen-bound phosphate influences glycogen architecture with different branching and chain length. Even if glucose phosphates are structurally similar, G1P and G6P are involved in the processes of glycogen degradation and synthesis, respectively. Oppositely, G2P or G3P residues cannot be confirmed by biochemical enzymatic assays as there is no known enzyme with specific activity towards these two phosphates [9,10,11]. Actually, glucose-2-phsophate itself was used for studying phosphate stability or hydrolysis rates [8,12,13], or for identification of the hydrolyzed monomeric forms of glycogen to understand the metabolism of the phosphate in this biopolymer [9,14]. The quite uncommon d-glucose-2-phosphate (G2P), which refer to phosphate covalently bonded glucose hydroxyl at C-2 position, was firstly and unequivocally synthesized by Farrar [15] in 1949. The hydrolysis of the cyclic glucose-1,2-phosphate (GCP) can afford a mixture of G2P and G1P. During hydrolysis, G2P esters exhibit higher stability versus G1P [12,15]. 

### 2.1. Phosphorylation and Coupling Methods

**Scheme 1:**
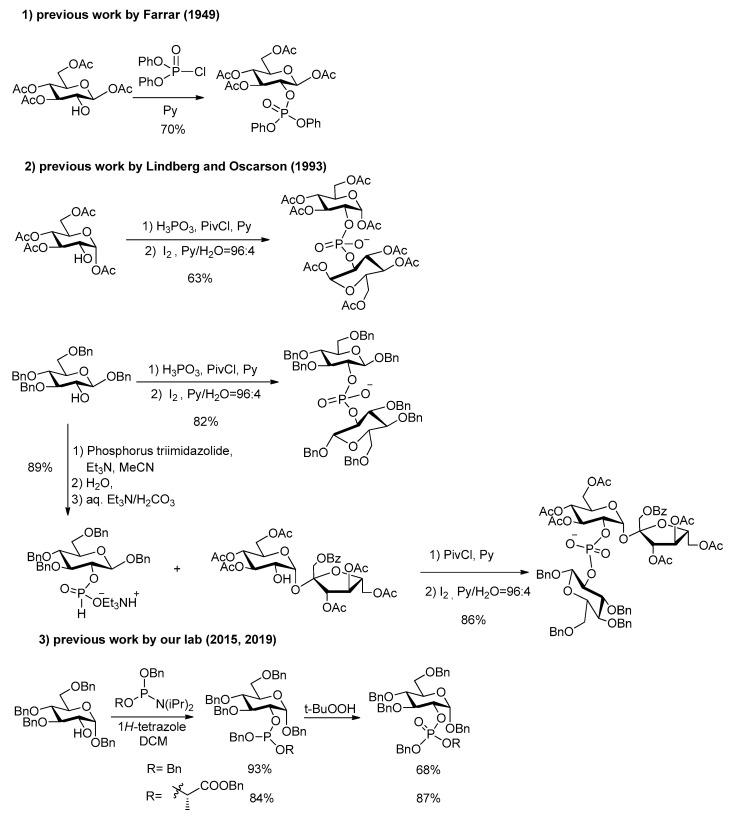
Previous synthesis of the precursors of agrocinopine C and D, G2P and G2LP.

In the synthesis of G2P and its phosphodiesters, a first issue is the choice between various methods for installing the phosphorus on the glucose residue and further connecting the other moiety. Several methods have been developed in the field of carbohydrates and oligonucleotide [16], glycoside and peptide synthesis, but only few have been applied to the case of G2P and its esters. Farrar’s first unequivocal G2P synthesis [15] involved diphenyl chlorophosphate (3 days in pyridine) as phosphorylation reagent (Scheme 1). The method used by Lindberg and Oscarson [17] for reaching an agrocinopine C precursor was the condensation of sucrose derivative and hydrogenphosphate in pyridine, while for reaching an agrocinopine D precursor, they performed the condensation of glucose derivative with a phosphonic acid in pyridine, activated by an acyl chloride [18,19]. The use of excess glucose building block was needed to make the reaction complete. The purification of the highly polar phosphonate diesters intermediates (in their salt form) requires consecutive ion exchange columns.

In the chemistry of agrocinopines A and B [20], which involve an arabinose building block instead of a glucose one in agrocinopines C and D, Thiem and coworkers used trichloroethyl phosphorodichloridate with 1,2,4-triazole in pyridine as coupling reagent. More recently, our team has used the convenient phosphoramidite strategy for constructing the phosphodiester both in the arabinose and glucose series [6,7]. This method combines easiness and flexibility for a diversity-oriented strategy towards a variety of agrocinopine analogs from various sugars and alcohols.

### 2.2. Building Blocks

**Scheme 2:**
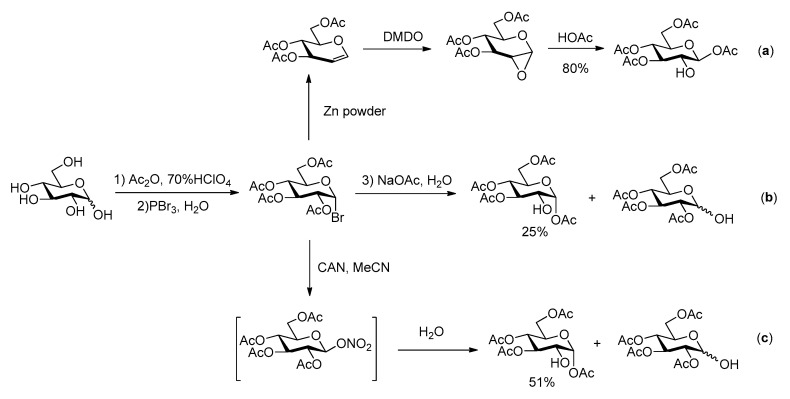
Previous synthetic routes for acetylated glucose with 2-OH free [21,22,23,24].

The overall efficiency of these syntheses relies also on the availability of the carbohydrate building blocks possessing the appropriate partial protection required for selective phosphorylation at C-2. Several 2-OH free glucoses have been reported in literature, most of them with benzyl and/or acetyl groups at *O*-1, 3, 4 and 6. In the synthesis of G2P, Farrar [15] used 1,3,4,6-tetraacetyl-β-glucose, which was prepared following Hardegger and Pascual procedure [21] by reaction of 3,4,6-tri-*O*-acetyl-1,2-anhydro-α-d-glucopyranose with HOAc (Scheme 2a). The easy purification by crystallization makes this sequence quite efficient. Alternatively, the hydrolysis of halogenated acetoglucose can also lead to tetraacetyl glucose with only 2-OH free [22,23,24] (Scheme 2b,c). 

**Scheme 3:**
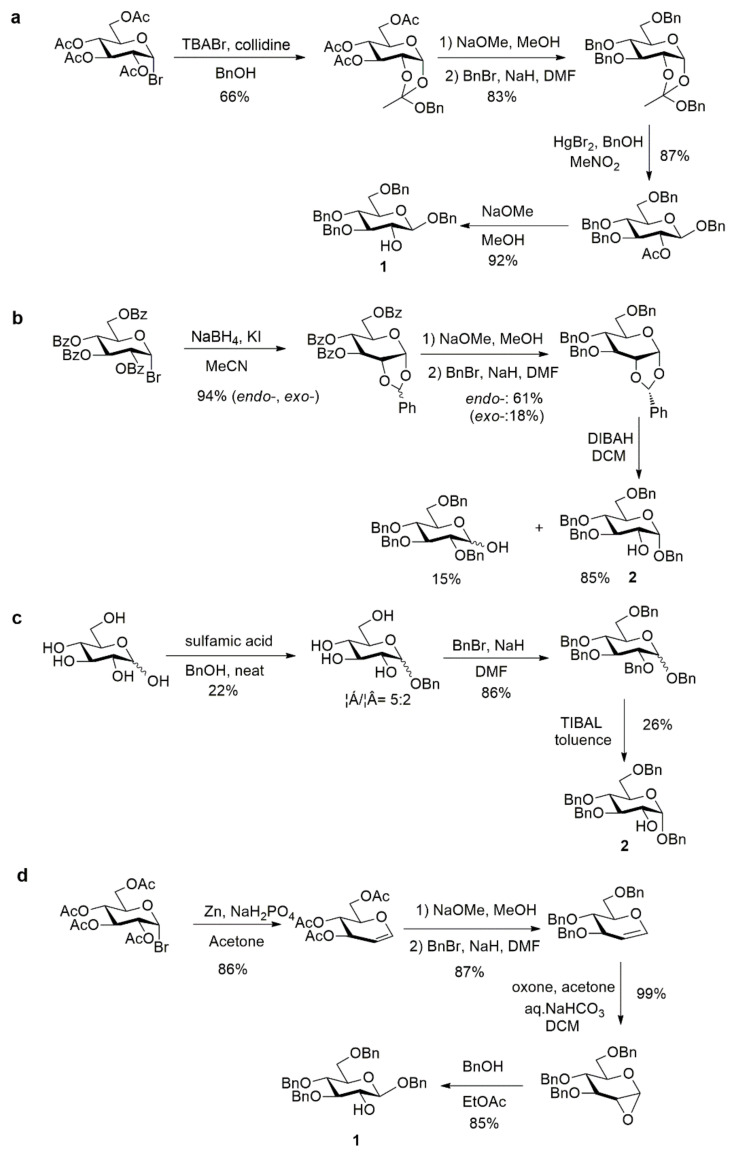
Various accesses to tetrabenzylated glucose with 2-OH free [26,27,28,29].

However, considering the risk of acetyl group migrations, leading to undesired mixtures of regioisomers in the course of the phosphorylation step, less labile protecting groups should be preferred. For example, one of us reported an allyl glucoside with only the 2-OH free as other positions are protected as a 4,6-*O*-benzylidene and a 3-*O*-allyl ether [25]. Even more appropriate is the use of benzyl ethers, both at the anomeric and other positions, in order to keep an easy final and practical deprotection procedure. Therefore, benzyl 3,4,6-*tri*-*O*-benzyl-d-glucopyranoside appears as the ideal glucose precursor. In the only previously reported synthesis of agrocinopine D, Lindberg and Oscarson [17] used this building block as its β anomer. They relied on a 5 steps synthesis starting from tetra-*O*-acetyl-α-d-glucopyranosyl bromide, formation of the 1,2-orthoester, Zemplén deacetylation followed by Williamson etherification using benzyl bromide to the tri-*O*-benzyl orthoester, then HgBr_2_-mediated glycosylation with benzyl alcohol and deacetylation to tetrabenzyl derivative **1** (Scheme 3a). The α anomer benzyl 3,4,6-*tri*-*O*-benzyl-α-d-glucopyranoside **2** can be made from *endo*-type 1,2-*O*-benzylidene acetal [26] or using the elegant reductive selective 2-*O-*debenzylation promoted by triisobutyl aluminum (TIBAL) [27] of perbenzyl glucose [6,7] (Scheme 3b,c). However, this latter debenzylation step only proceeds on the α anomer, limiting significantly the overall yield when mixtures of anomers are used as substrate as all the β anomer remains untransformed. The shortest and most efficient sequence appears to be based on the Lewis acid-catalyzed opening reaction of the readily available 1,2-anhydro-α-d-glucopyranose [28,29] leading to **1** in high yield within only 2 steps from the commercially available benzylated glucal (Scheme 3d). Based on this survey, this latter access to **1** was used in the following work towards G2P and G2P esters.

### 2.3. Alternative Flexible Access to G2P and Esters

Aiming at proposing a more general and flexible access able to lead to G2P or its esters, we thus applied the phosphoramidite coupling strategy to the 3,4,6-tri-*O*-benzyl-β-d-glucopyranoside building block **1**. Reaction with benzyloxy bis(diisopropylamino)phosphine gave selectively the monoaminated phosphoramidite **3** in 81% yield in the presence of diisopropylammonium tetrazolide as mild and hindered base preventing double diisopropylamino displacement [30]. The phosphoramidite **3** was then used in coupling reactions with different alcohols, as shown in Table 1. 

The conversion of phosphoramidite **3** into phosphotriesters **4** was conducted in a two-step one-pot procedure, involving firstly the reaction with the alcohol leading to an intermediate phosphite, and secondly the oxidation of the phosphite to the phosphate using *tert*-BuOOH. The reaction with benzyl alcohol gave the fully protected G2P **4a** in 88% yield. The reaction was also conducted with a short scope of primary or secondary alcohols including cetearyl alcohol, cholesterol, 5α-cholestan-3β-ol (dihydrocholesterol) and glucoside **1** having only 2-OH available. All gave fair to good yields, with primary alcohols showing logically higher reactivity, while secondary suffer from steric hindrance. Owing to the chiral center of the phosphorus atom, phosphates **4b**, **4c** and **4d** were obtained as a mixture of the two diastereoisomers in nearly 1:1 ratio determined by ^31^P-NMR spectra. 

**Scheme 4:**
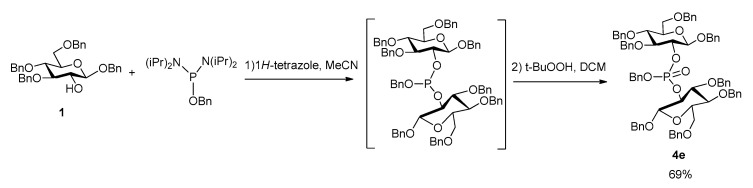
Synthesis of the precursor of agrocinopine D in one-pot.

Considering the interest of agrocinopine D, and its symmetrical structure, we investigated a one-pot access to the protected phosphodiester **4e** from the glucose building block **1** in which the intermediate bis-glucosyl phosphite was not isolated (Scheme 4). This step was optimized with respect to the stoichiometry between substrate **1**, phosphoramidite and tetrazole. When the reaction was carried out with only 1.0 equivalent phosphoramidite (compared to the 2.0 equivalents of substrate **1**), low conversion of substrate **1** was observed. Increasing the amount of phosphoramidite to 2.0 equivalents led mainly to the formation of monoaminated phosphoramidite **3**. The most favorable conditions were found to be 1.2 equivalents of phosphoramidite and 3 equivalents of 1*H*-tetrazole.

**Scheme 5:**
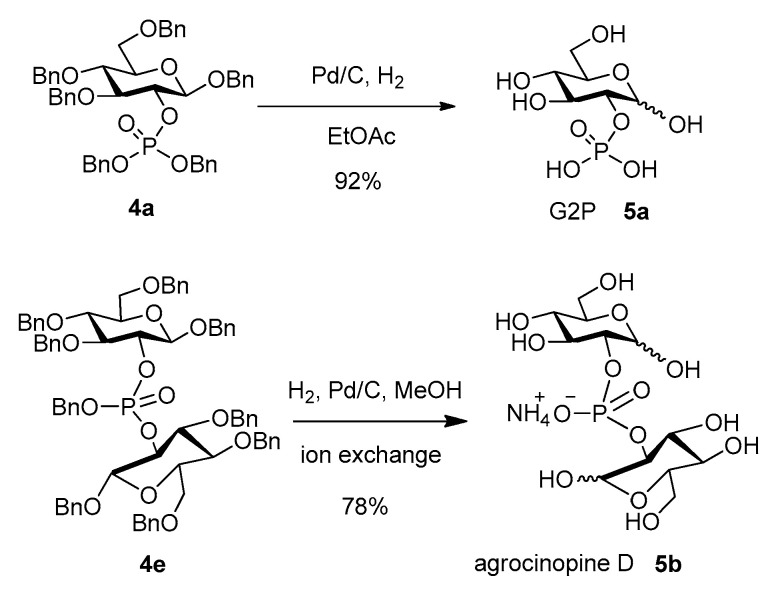
Deprotection of the phosphate precursors.

The fully deprotected targets G2P (**5a**) and agrocinopine D (**5b**) were obtained after palladium-catalyzed hydrogenolysis from compounds **4a** and **4e** respectively, using 1 atm H_2_ and Pd/C in methanol (Scheme 5). G2P **5a** was obtained in high yield and purity after debenzylation in ethyl acetate. Agrocinopine D **5b** was obtained in 78% yield as its ammonium salt after ion exchange chromatography on a DEAE-Sephadex column. Both compounds exhibited identical data as compared to literature [6,17].

## 3. Materials and Methods

### 3.1. General Methods

All commercial reagents and solvents were used without further purification. Analytical thin layer chromatography was performed on aluminum-backed plates coated with Macherey-Nagel Kieselgel 60 XtraSIL G/UV254. Macherey-Nagel Kieselgel 60 M silica was used for flash chromatography. Synthetic products were visualized under UV light (at 254 nm) or stained using concentrated sulfuric acid. NMR spectra were recorded on a Bruker 300 MHz or a Bruker 500 MHz spectrometer. Chemical shifts (ppm) were reported relative to residual solvent signals. Multiplicities are abbreviated as follows: s, singlet; d, doublet; t, triplet; m, multiplet. Coupling constants were measured in Hertz (Hz). High-resolution mass spectra (HRMS) were performed by a Finnigan Mat 151xL mass spectrometer using electrospray (see supplementary materials).

### 3.2. Synthesis of benzyl 3,4,6-tri-O-benzyl-2-O-[benzyloxy(diisopropylamino)phosphanyl]-β-D-glucopyranoside (or benzyl ((2R,3R,4S,5R,6R)-2,4,5-tris(benzyloxy)-6-((benzyloxy)methyl)tetrahydro-2H-pyran-3-yl) diisopropylphosphoramidite) ***3***

Benzyl 3,4,6-tri-*O*-benzyl-β-d-glucopyranoside (**1**) was synthesized from commercially available 3,4,6-tri-*O*-benzyl-d-glucal as previously reported [26,27]. After recrystallization from ethanol, the resulting product **1** as white solid was subjected to the next step. To a solution of diisopropylammonium tetrazolide salt, prepared by mixing tetrazole (1.0 eq, 4.2 mL of a 0.45M solution in MeCN) with diisopropylamine (1.2 eq, 0.33 mL) and benzyloxy bis(diisopropylamino)phosphine (0.96 g, 2.7 mmol, 1.5 eq.) in 10 mL DCM, was added benzyl 3,4,6-tri-*O*-benzyl-β-d-glucopyranoside (**1**, 1 g, 1.8 mmol), which was dissolved in 10 mL DCM at 0 °C. Then the mixture was stirred overnight at room temperature. The reaction was monitored by TLC (EtOAc-pentane 1:9 with 1% Et_3_N as eluent. When the starting material was consumed, the solid salt was filtered. The organic phase was collected, concentrated and purified by silica gel chromatography (EtOAc-pentane 1:9 with 1% Et_3_N) to give 1.2 g of the desired product (yield 81%). Rf = 0.56. ^1^H-NMR (CDCl_3_, 500 MHz): δ (ppm) 7.38–7.09 (m, 23H, Ph), 7.07–7.00 (m, 2H, Ph), 4.93 (m, 1H, C***H***HPh), 4.84 (m, 1H, C***H***HPh), 4.74–4.43 (m, 8H, 4×C**H_2_**Ph), 4.40 (d, *J* = 7.5 Hz, 1H, H-1), 3.84 (m, 1H, H-2), 3.66–3.51 (m, 6H, H6a, H6b, H-4, H-3, 2×CH), 3.42 (m, 1H, H-5), 1.07–0.96 (m, 12H, 4×CH_3_); ^13^C-NMR (126 MHz, CDCl_3_) δ (ppm) 139.9 (C-Ph), 139.6 (C-Ph), 138.8 (C-Ph), 138.3 (C-Ph), 137.7 (C-Ph), 128.4 (Ph), 128.4 (Ph), 128.4 (Ph), 128.3 (Ph), 128.2 (Ph), 128.2 (Ph), 128.1 (Ph), 128.0 (Ph), 128.0 (Ph), 128.0 (Ph), 127.9 (Ph), 127.8 (Ph), 127.8 (Ph), 127.8 (Ph), 127.7 (Ph), 127.7 (Ph), 127.6 (Ph), 127.6 (Ph), 127.6 (Ph), 127.4 (Ph), 127.3 (Ph), 127.2 (Ph), 127.1 (Ph), 127.0 (Ph), 127.0 (Ph), 102.0 (C1), 101.7 (d, *J*_C-P_ = 2.9 Hz, C1′), 85.5 (d, *J*_C-P_ = 2.8 Hz, C3), 85.4 (C3′), 78.1 (C4), 76.2 (d, *J*_C-P_ = 10.9 Hz, C2’), 75.9 (d, *J*_C-P_ = 5.2 Hz, C2), 75.2 (**C**H_2_Ph), 75.0 (C5, C5’), 74.9 (**C**H_2_Ph), 73.5 (**C**H_2_Ph), 70.8 (**C**H_2_Ph), 69.2 (C6, C6′), 65.5 (**C**H_2_Ph), 43.1 (CH), 43.0 (CH), 24.7 (CH_3_), 24.6 (CH_3_), 24.6 (CH_3_), 24.5 (CH_3_). ^31^P-NMR (CDCl_3_,122 MHz): δ (ppm) 151.44, 150.23 (ratio 1/1.3). HR-MS (ESI): C_47_H_59_NO_8_P [M + H_3_O]^+^ requires: 796.3973; found: 796.3976.

### 3.3. General Procedure for the two-step Synthesis of Phosphotriesters ***4a**–**e***

To a solution of phosphoramidite **3** (155 mg**,** 0.2 mmol) in 2 mL DCM was added corresponding alcohol (0.2 mmol) dissolved in 1 mL DCM, 1*H*-tetrazole (4.5 mL of a 0.45 M solution in MeCN, 0.2 mmol) at rt. Then the mixture was stirred for 5 h at rt. After completion of the reaction, *t*-BuOOH (43 µL of a 5.6 M solution in decane, 0.24 mmol) was added. After 1 h, the white solid was filtered. The organic phase was collected, concentrated and purified by silica gel chromatography.

#### 3.3.1. Benzyl 3,4,6-tri-*O*-benzyl-2-*O-*dibenzylphosphonato-β-D-glucopyranoside [or dibenzyl ((2*R,*3*R,*4*S*,5*R,*6*R*)-2,4,5-tris(benzyloxy)-6-((benzyloxy)methyl)tetrahydro-2*H*-pyran-3-yl) phosphate] **4a**

From benzyl alcohol (20 μL) following general procedure, TLC monitoring using EtOAc-pentane 1:3 as eluent, flash chromatography of the crude product (1:3 EtOAc-pentane) afforded **4a** (yield 88%) as a white solid. Rf = 0.43. ^1^H-NMR (CDCl_3_, 500 MHz): δ (ppm) 7.31 (m, 8H, Ph), 7.25–7.13 (m, 16H, Ph), 7.12–7.01 (m, 6H, Ph), 4.93–4.85 (m, 4H, 2×C**H_2_**Ph), 4.82 (m, 1H, C***H***HPh), 4.79–4.68 (m, 3H, C***H***HPh, C**H_2_**Ph), 4.61–4.55 (m, 2H, C**H_2_**Ph), 4.51 (d, *J* = 10 Hz, 1H, H1), 4.50–4.47 (m, 2H, C**H_2_**Ph), 4.45–4.39 (m, 1H, H2), 3.70–3.62 (m, 4H, H6a, H6b, H3, H4), 3.45 (m, 1H, H5); ^13^C-NMR (CDCl_3,_ 126 MHz): δ (ppm) 138.3, 138.1, 137.9, 136.9, 136.2, 136.1, 128.4, 128.4, 128.4, 128.4, 128.3, 128.2, 128.2, 128.0, 127.9, 127.8, 127.8, 127.7, 127.7, 127.7, 127.6, 127.5, 100.1 (d, *J*_C-P_ = 3.7 Hz, C1), 83.7 (d, *J*_C-P_ = 4.0 Hz, C3), 79.1 (d, *J*_C-P_ = 6.9 Hz, C2), 77.8 (C4), 75.2 (C5), 75.1 (O**C**H_2_Ph), 75.0 (O**C**H_2_Ph), 73.6 (O**C**H_2_Ph), 70.8 (O**C**H_2_Ph), 69.2 (d, *J*_C-P_ = 5.2 Hz, O**C**H_2_Ph), 69.1 (d, *J*_C-P_ = 5.3 Hz, O**C**H_2_Ph), 68.7 (C6); ^31^P-NMR (CDCl_3,_ 122 MHz,): δ (ppm) −1.71. [α${]}_{D}^{24}$ = −25.1 (c = 0.4, acetone). mp: 83.1~83.4 °C. HR-MS (ESI): C_48_H_49_NaO_9_P [M + Na]^+^ requires: 823.3006; found: 823.3006.

#### 3.3.2. Benzyl 3,4,6-tri-*O*-benzyl-2-*O*-[(benzyl)(hexadecyl)phosphonato]-β-D-glucopyranoside [or benzyl hexadecyl ((2*R,*3*R,*4*S*,5*R,*6*R*)-2,4,5-tris(benzyloxy)-6-((benzyloxy)methyl)tetrahydro-2*H*-pyran-3-yl) phosphate] **4b**

From cetearyl alcohol (48 mg) following general procedure, TLC monitoring using EtOAc-pentane 1:3 as eluent, flash chromatography of the crude product (1:1 EtOAc-pentane) afforded **4b** (yield 90%) as a white solid. Rf = 0.4. ^1^H-NMR (CDCl_3_, 500 MHz): δ (ppm) 7.46–7.41 (m, 1H, Ph), 7.41–7.12 (m, 24H, Ph), 5.08–4.88 (m, 4H, 2×CH_2_Ph), 4.88–4.81 (m, 1H, CHHPh), 4.81–4.75 (m, 1H, CHHPh), 4.70–4.61 (m, 2H, CH_2_Ph), 4.57 (d, *J* = 8.3 Hz, 1H, H1), 4.56–4.53 (m, 2H, CH_2_Ph), 4.45 (m, 1H, H2), 3.94–3.81 (m, 2H, OCH_2_), 3.79–3.68 (m, 4H, H3, H4, H6a, H6b), 3.51 (m, 1H, H5), 1.51–1.37 (m, 2H, CH_2_), 1.28 (m, 26H, 13×CH_2_), 0.90 (t, *J* = 6.9 Hz, 3H, CH_3_); ^13^C-NMR (CDCl_3_, 126 MHz): δ (ppm) 138.3 (Ph), 138.1 (Ph), 137.9 (Ph), 137.0 (Ph), 136.3 (Ph), 128.6 (Ph), 128.5 (Ph), 128.4 (Ph), 128.4 (Ph), 128.4 (Ph), 128.3 (Ph), 128.3 (Ph), 128.3 (Ph), 128.2 (Ph), 128.1 (Ph), 128.1 (Ph), 128.0 (Ph), 128.0 (Ph), 127.9 (Ph), 127.8 (Ph), 127.8 (Ph), 127.8 (Ph), 127.7 (Ph), 127.7 (Ph), 127.6 (Ph), 127.6 (Ph), 127.4 (Ph), 100.1 (d, *J*_C-P_ = 3.6 Hz, C1, C1’), 83.7 (t, *J*_C-P_ = 3.6 Hz, C3, C3’), 78.9 (t, *J*_C-P_ = 7.5 Hz, C2, C2’), 77.8 (C4, C4’), 75.2 (C5, C5’), 75.0 (OCH_2_Ph), 73.5 (OCH_2_Ph), 70.8 (OCH_2_Ph), 69.2 (d, *J*_C-P_ = 8.2 Hz, OCH_2_Ph), 69.1 (d, *J*_C-P_ = 5.4 Hz, OC’H_2_Ph), 69.0 (OCH_2_Ph), 68.7 (C6, C6′), 68.2 (d, *J*_C-P_ = 10.3 Hz, OCH_2_), 68.2 (d, *J*_C-P_ = 6.0 Hz, OC’H_2_), 32.0, 30.1, 29.8, 29.7, 29.7, 29.7, 29.6, 29.5, 29.4, 29.1, 25.4, 25.3, 22.7, 14.2; ^31^P-NMR (CDCl_3_,122 MHz): δ (ppm) −1.55, −1.80 (ratio 1/1.1). HR-MS (ESI): C_57_H_75_NaO_9_P [M + Na]^+^ requires: 957.5041; found: 957.5038.

#### 3.3.3. Benzyl 3,4,6-tri-*O*-benzyl-2-*O*-[(benzyl)(cholesteryl)phosphonato]-β-D-glucopyranoside [or benzyl cholesteryl ((2*R*,3*R,*4S,5*R,*6*R*)-2,4,5-tris(benzyloxy)-6-((benzyloxy)methyl)tetrahydro-2*H*-pyran-3-yl) phosphate] **4c**

From cholesterol (79 mg) following general procedure, TLC monitoring using EtOAc-pentane 1:3 as eluent, flash chromatography of the crude product (1:4 EtOAc-pentane) afforded **4c** (yield 70%) as a white solid. Rf = 0.56. ^1^H-NMR (CDCl_3_, 500 MHz): δ (ppm) 7.35–7.00 (m, 25H, Ph), 5.12 (m, 1H, C=CH-Cholesterol), 4.98–4.79 (m, 4H, 2×C**H_2_**Ph), 4.78–4.66 (m, 2H, C**H_2_**Ph), 4.62–4.52 (m, 2H, C**H_2_**Ph), 4.47 (d, *J* = 6 Hz, 1H, H1), 4.46–4.42 (m, 2H, C**H_2_**Ph), 4.34 (m, 1H, H2), 4.05 (m, 1H, OCH-Chol), 3.69–3.57 (m, 4H, H3, H4, H6a, H6b), 3.41 (m, 1H, H5), 2.34–2.11 (m, 2H, CH_2_-Chol), 1.98–1.65 (m, 5H, Chol), 1.63–1.12 (m, 12H, Chol), 1.11–0.63 (m, 21H, Chol), 0.60–0.53 (m, 3H, Chol); ^13^C-NMR (CDCl_3_, 126 MHz): δ (ppm) 139.5 (**C**=CH, Chol), 138.4, 138.1, 137.9, 137.1, 136.4, 128.6, 128.4, 128.3, 128.2, 128.1, 128.0, 127.9, 127.8, 127.7, 127.6, 127.4, 122.7 (C=**C**H, Chol), 100.2 (d, *J*_C-P_ = 3.5 Hz, C1, C1’), 83.7 (d, *J*_C-P_ = 3.7 Hz, C3), 78.9 (t, *J*_C-P_ = 6.7 Hz, C2, C2’), 78.6 (d, *J*_C-P_ = 6.0 Hz, OCH-Chol), 78.5 (d, *J*_C-P_ = 5.8 Hz, OC’H-Chol), 77.8 (C4, C4’), 75.1 (C5, C5’), 75.0 (O**C**H_2_Ph), 74.9 (O**C**H_2_Ph), 73.5 (O**C**H_2_Ph), 70.8 (O**C**H_2_Ph), 69.0 (d, *J*_C-P_ = 5.5 Hz, O**C**H_2_Ph), 68.7 (C6, C6’), 56.7, 56.2, 49.8, 42.3, 39.8, 39.6, 36.7, 36.6, 36.3, 36.2, 35.8, 31.9, 31.8, 29.4, 29.3, 28.3, 28.1, 24.3, 23.9, 22.9, 22.6, 21.0, 19.3, 19.2, 18.8, 11.9. ^31^P-NMR (CDCl_3_, 202 MHz): δ (ppm) −2.60, −2.98 (ratio 1/1.3). HR-MS (ESI): C_68_H_88_O_9_P [M + H]^+^ requires: 1079.6160; found: 1079.6170.

#### 3.3.4. Benzyl 3,4,6-tri-*O*-benzyl-2-*O-*[(benzyl)(5α-cholestan-3β-yl)phosphonato]-β-D-glucopyranoside [or benzyl cholestanyl ((2*R*,3*R*,4*S*,5*R*,6*R*)-2,4,5-tris(benzyloxy)-6-((benzyloxy)methyl)tetrahydro-2*H*-pyran-3-yl) phosphate **4d**

From 5α-cholestan-3β-ol (79 mg) following general procedure, TLC monitoring by TLC using EtOAc-pentane 1:3 as eluent, flash chromatography of the crude product (1:3 EtOAc-pentane) afforded **4d** (yield 74%) as a white solid. Rf = 0.37. ^1^H-NMR (CDCl_3_, 500 MHz): δ (ppm) 7.46–7.09 (m, 25H, Ph), 4.97 (m, 3H, C***H***HPh, C**H_2_**Ph), 4.92–4.74 (m, 3H, C***H***HPh, C**H_2_**Ph), 4.70–4.61 (m, 2H, C**H_2_**Ph), 4.57–4.56 (m, 2H, C**H_2_**Ph), 4.54 (d, *J* = 8.3 Hz, 1H, H1), 4.45–4.40 (m, 1H, H2), 4.23–4.09 (m, 1H, OCH-Chol), 3.81–3.66 (m, 4H, H3, H4, H6a, H6b), 3.51 (m, 1H, H5), 1.95 (m, 1H, Chol), 1.86–1.73 (m, 2H, Chol), 1.63–1.50 (m, 4H, Chol), 1.44 (d, *J* = 7.4 Hz, 1H, Chol), 1.41–0.94 (m, 20H, Chol), 0.93–0.84 (m, 9H, Chol), 0.82–0.70 (m, 2H, Chol), 0.69–0.60 (m, 6H, Chol), 0.55–0.39 (m, 1H, Chol); ^13^C-NMR (CDCl_3_, 126 MHz): δ (ppm) 138.4, 138.1, 137.9, 137.2, 136.5, 128.4, 128.3, 128.3, 128.3, 128.3, 128.3, 128.0, 128.0, 128.0, 127.9, 127.8, 127.8, 127.8, 127.8, 127.7, 127.7, 127.7, 127.5, 127.4, 100.2 (d, *J*_C-P_ = 1.9 Hz, C1, C1’), 83.7 (d, *J*_C-P_ = 3.7 Hz, C3, C3’), 78.9 (d, *J*_C-P_ = 7.2 Hz, C2), 78.8 (d, *J*_C-P_ = 7.0 Hz, C2′), 78.6 (d, *J*_C-P_ = 6.0 Hz, O**C**H-Chol), 77.8 (C4, C4’), 75.1 (C5, C5’), 75.0 (O**C**H_2_Ph), 74.7 (O**C**H_2_Ph), 73.5 (O**C**H_2_Ph), 70.8 (O**C**H_2_Ph), 69.0 (d, *J*_C-P_ = 5.4 Hz, O**C**H_2_Ph), 68.7 (C6), 56.5, 56.3, 54.0, 44.3, 42.6, 40.0, 39.6, 36.6, 36.2, 35.8, 35.6, 35.1, 32.0, 29.3, 29.1, 28.5, 28.3, 28.1, 24.2, 23.9, 22.9, 22.6, 21.2, 18.7, 12.2, 12.1. ^31^P-NMR (CDCl_3_,122 MHz) δ (ppm) −2.47, −2.85 (ratio 1/1.4). HR-MS (ESI): C_68_H_90_O_9_P [M + H]^+^ requires: 1081.6317; found: 1081.6309.

#### 3.3.5. Benzyl 3,4,6-tri-*O*-benzyl-2-*O*-[(benzyl)[(1,3,4,6-tetrabenzyl-β-d-glucopyranos-2-yl)phosphonato]-β-d-glucopyranoside [or benzyl bis((2*R*,3*R*,4*S*,5*R*,6*R*)-2,4,5-tris(benzyloxy)-6-((benzyloxy)methyl)tetrahydro-2*H*-pyran-3-yl) phosphate] **4e**

From compound **1** (108 mg) following general procedure, TLC monitoring using EtOAc-pentane 1:3 as eluent, flash chromatography of the crude product (1:3 EtOAc-pentane) afforded **4e** (yield 53%) as a white solid. Rf = 0.36. ^1^H-NMR (CDCl_3_ 500 MHz): δ (ppm) 7.49–7.41 (m, 4H, Ph), 7.37–7.27 (m, 26H, Ph), 7.25–7.12 (m, 13H, Ph), 7.07 (m, 2H, Ph), 5.01–4.84 (m, 6H, 3×C**H_2_**Ph), 4.73 (d, *J* = 10.8 Hz, 2H, C**H_2_**Ph), 4.71–4.66 (m, 2H, C**H_2_**Ph), 4.66–4.45 (m, 8H, 4×C**H_2_**Ph), 4.44–4.39 (m, 1H, H2), 4.35 (m, 1H, H2′), 4.30 (d, *J* = 6.9 Hz, 1H, H1), 4.29 (d, *J* = 6.9 Hz, 1H, H1′), 3.68 (m, 2H, H6), 3.61 (m, 2H, H6′), 3.53–3.36 (m, 4H, H4, H4′, H3, H3′), 3.35–3.26 (m, 2H, H5, H5′); ^13^C-NMR (CDCl_3_,126 MHz): δ (ppm) 138.5, 138.2, 138.0, 137.3, 137.3, 128.4, 128.4, 128.3, 128.3, 128.3, 128.2, 128.2, 127.9, 127.9, 127.8, 127.8, 127.8, 127.7, 127.7, 127.7, 127.6, 127.5, 127.5, 127.4, 127.4, 100.2 (d, *J*_C-P_ = 2.9Hz, C1′), 99.8 (d, *J*_C-P_ = 4.2 Hz, C1), 83.6 (d, *J*_C-P_ = 4.8 Hz, C3′), 83.5 (d, *J*_C-P_ = 3.6 Hz, C3), 79.1 (d, *J*_C-P_ = 7.4 Hz, C2′), 78.6 (d, *J*_C-P_ = 6.8 Hz, C2), 77.7 (C4’), 77.6 (C4), 75.0 (C5), 74.8 (C5’), 74.8 (O**C**H_2_Ph), 74.8 (O**C**H_2_Ph), 74.7 (O**C**H_2_Ph), 74.7 (O**C**H_2_Ph), 73.4 (O**C**H_2_Ph), 73.4 (O**C**H_2_Ph), 70.9 (O**C**H_2_Ph), 70.2 (O**C**H_2_Ph), 69.3 (d, *J*_C-P_ = 5.0 Hz, O**C**H_2_Ph), 68.7 (C6, C6′); ^31^P-NMR (CDCl_3_,122 MHz) δ (ppm) −3.64. HR-MS (ESI): C_75_H_77_NaO_14_P [M + Na]^+^ requires: 1255.4943; found: 1255.4949.

### 3.4. Procedure for the one-step Synthesis of **4e**

To a solution of benzyl 3,4,6-tri-*O*-benzyl-β-d-glucopyranoside (**1**) (783 mg, 1.45 mmol, 2 eq.) and benzyloxy bis(diisopropylamino)phosphine (297 mg, 0.88 mmol, 1.2 eq.) in 9 mL of acetonitrile was added 1*H*-tetrazole (4.95 mL of a 0.45 solution in MeCN, 2.2 mmol, 3 eq.) at rt. Then the mixture was stirred overnight at rt. After full conversion of the starting glucoside (TLC using EtOAc-pentane 1:4.5 as eluent, intermediate phosphite Rf = 0.3), the mixture was cooled to 0 °C, then *t*-BuOOH (170 μL, 0.88 mmol, 1.2 eq.) dissolved in 15 mL of DCM was added and the reaction mixture was allowed to warm to rt. After 4 h, TLC using EtOAc-pentane 1:3 as eluent indicated that the product **4e** (Rf = 0.36) was formed and no starting material remained. The white solid was filtered, the organic phase was collected, concentrated and purified by silica gel chromatography, same as in previous section.

### 3.5. Procedure for the Debenzylation of Phosphodiesters ***4a*** and ***4e***

#### 3.5.1. d-Glucose-2-phosphate (G2P) **5a**

To a solution of phosphate **4a** (100 mg, 0.12 mmol) in 5 mL of EtOAc was added 10% Pd/C (60 mg). The mixture was stirred under 1 atm H_2_ at rt. After 24 h, Pd/C was filtered with a celite layer. The organic phase was concentrated to afford G2P as a white solid, which is showing high purity as seen by NMR(90% yield, α/β = 5:3). ^1^H-NMR (D_2_O, 300 MHz) δ (ppm) 5.33 (d, *J* = 3.3 Hz, 1H, H1α), 4.69 (d, *J* = 7.6 Hz, 0.6H, H1β), 3.98–3.88 (m, 1H, H2α), 3.86–3.59 (m, 5.8H, H2β, H3α, H5α, H6α, H6β), 3.58 (t, *J* = 7.7 Hz, 0.6H, H3β), 3.47–3.36 (m, 2.2H, H4α, H4β, H5β). NMR data were consistent with the reported [6].

#### 3.5.2. Agrocinopine D **5b**

To a solution of phosphate **4e** (100 mg, 0.08 mmol) in 5 mL of methanol was added 10% Pd/C (60 mg). The mixture was stirred under 1 atm H_2_ at rt for 1 day. TLC monitoring using DCM, acetone, MeOH, H_2_O 4:3:3:1 (vol.) shows formation of **5b** (Rf = 0.48). After filtration with a celite layer, the organic phase was concentrated to afford a white solid, which was further purified by ion exchange chromatography on a DEAE-Sephadex column pre-washed with water. The column was then eluted with a 5% to 100% gradient 1M ammonium carbonate. Freeze-drying the resulting fractions allows to get **5b** as a white solid in 78% yield. ^1^H-NMR (D_2_O, 500 MHz) δ (ppm) 5.39 (d, *J* = 3.6 Hz, 1H, H1α), 4.75 (d, *J* = 7.9 Hz, 0.5H, H1β), 4.09–3.96 (m, 1H, H2α), 3.93–3.60 (m, 6H, H2β, H3α, H3β, H5α, H6α, H6β), 3.51–3.43 (m, 2H, H4α, H4β, H5β). HR-MS (ESI) [M − H]^−^: 421.0753; found: 421.0735. NMR data were consistent with the reported [17]. HR-MS (ESI): C_12_H_22_O_14_P [M − H]^−^ requires: 421.0753; found: 421.0735.

## 4. Conclusions

A short and flexible access to the rare glucose-2-phosphate G2P esters including agrocinopine D is proposed. It relies on the combination of the use of benzyl 3,4,6-tri-*O*-benzyl-β-d-glucopyranoside prepared in two steps from the commercially available tri-*O*-benzyl-d-glucal and the phosphoramidite coupling strategy. It is applied to short and easy accesses to G2P itself and agrocinopine D.

## Figures and Tables

**Table 1:**
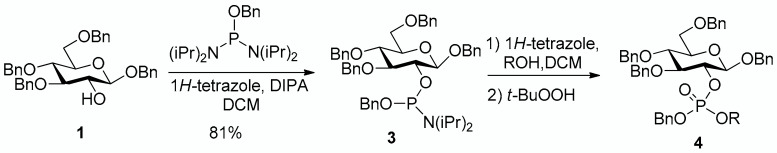
Synthesis of the precursors of glucose-2-phosphate and derivatives.

Entry	ROH	Product 4	Yield (%) (Diastereomeric Ratio) ^a^
1	benzyl alcohol	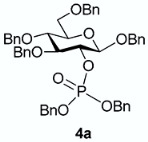	88
2	cetearyl alcohol	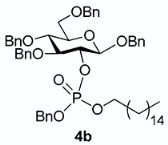	90 (1/1.1)
3	cholesterol	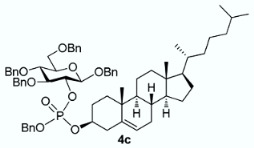	70 (1/1.3)
4	5α-cholestan-3β-ol	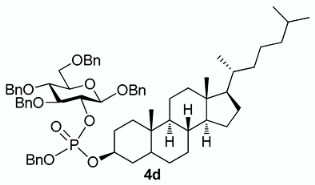	74 (1/1.4)
5	benzyl 3,4,6-tri-*O*-benzyl-β-d-glucopyranoside	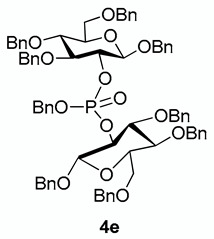	53

a. Measured by ^31^P-NMR.

## Supplementary Materials

The following are available online, NMR spectra for all new compounds.
