# Supplementary material for: Esters of Glucose-2-Phosphate: Occurrence and Chemistry
Source: Molecules. 2020 Jun 19;25(12):2829. doi: 10.3390/molecules25122829 (PMC7356202; doi:10.3390/molecules25122829)
Supplement: Supplementary file 1 [file molecules-25-02829-s001.pdf]

Esters of glucose-2-phosphate: occurrence and chemistry

Qiang Zhang, Si-zhe Li, Mohammed Ahmar, Laurent Soulère, Yves Queneau*

Univ Lyon, Université Claude Bernard Lyon 1, INSA Lyon, CPE Lyon, UMR 5246, CNRS, ICBMS, Institut de Chimie et de Biochimie Moléculaires et Supramoléculaires, Chimie Organique et Bioorganique, Bât. E. Lederer, 1 rue Victor Grignard F-69622 Villeurbanne, France.

Supplementary data – ^{1}H NMR, ^{13}C NMR and ^{31}P NMR spectra

---

* Corresponding authors at: ICBMS, Chimie Organique et Bioorganique, Bât. E. Lederer, 1 rue Victor Grignard F-69622 Villeurbanne, France.
E-mail addresses: yves.queneau@insa-lyon.fr

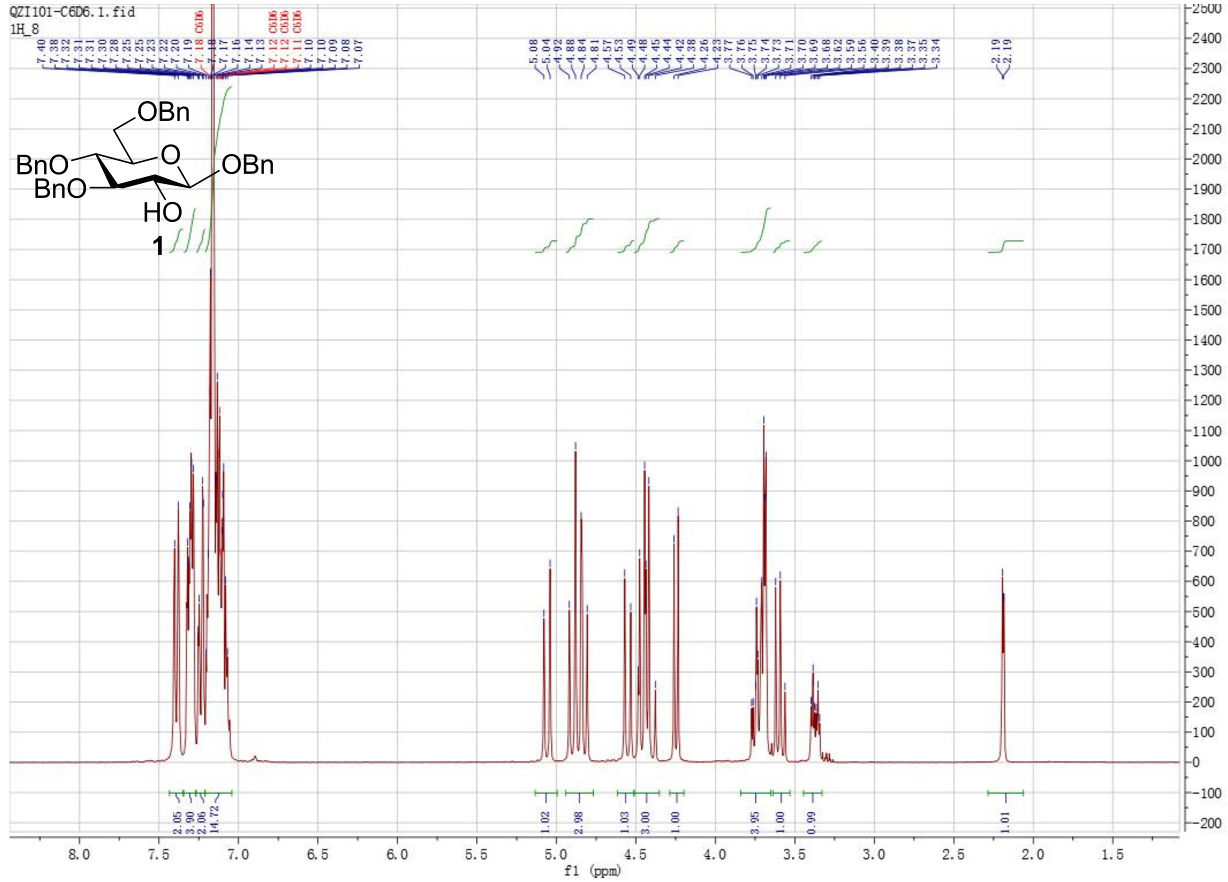
QZI101-C6D6.1.fid
1H_8
OBn
BnO
BnO
O
OBn
HO
1
f1 (ppm)

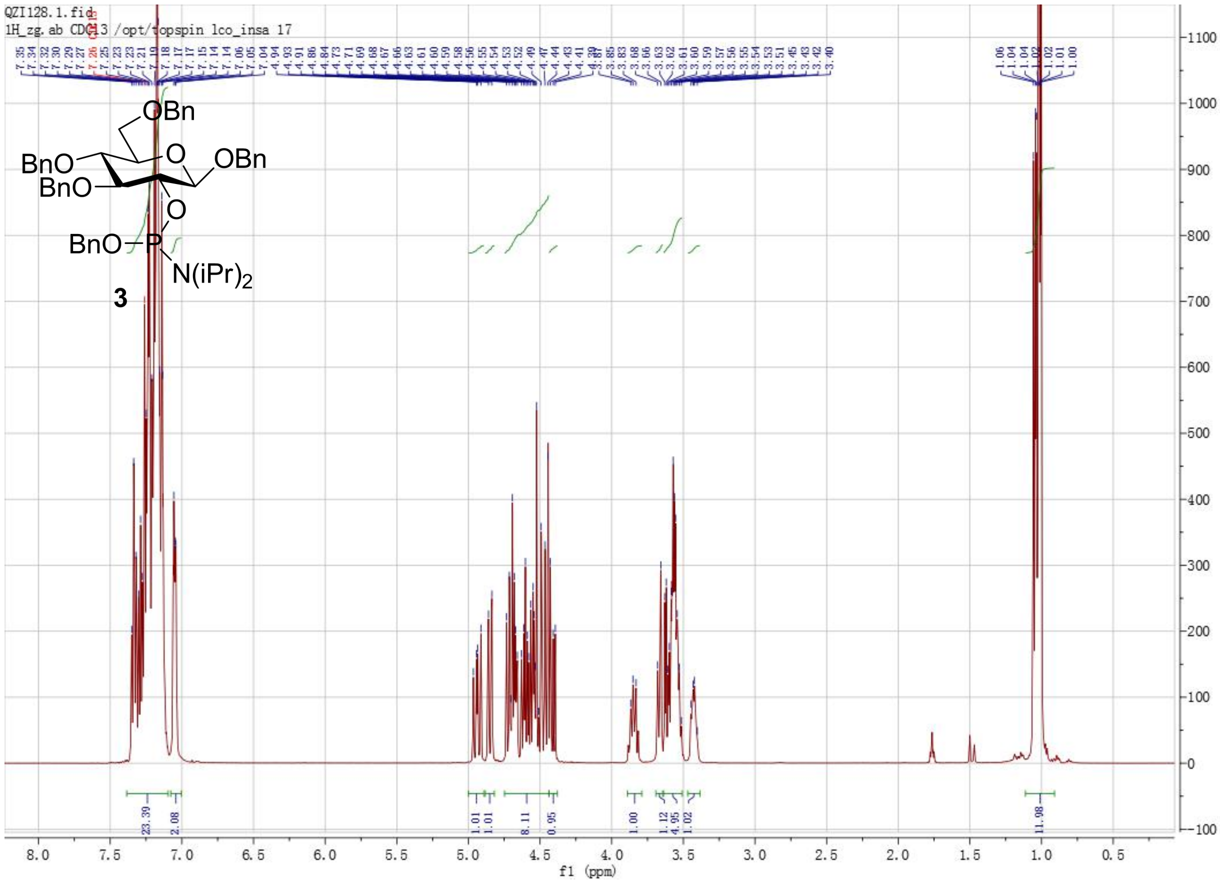
QZI128.1.fid
OBn
BnO
BnO
O
OBn
O
BnO
P
N(iPr)2
3
f1 (ppm)

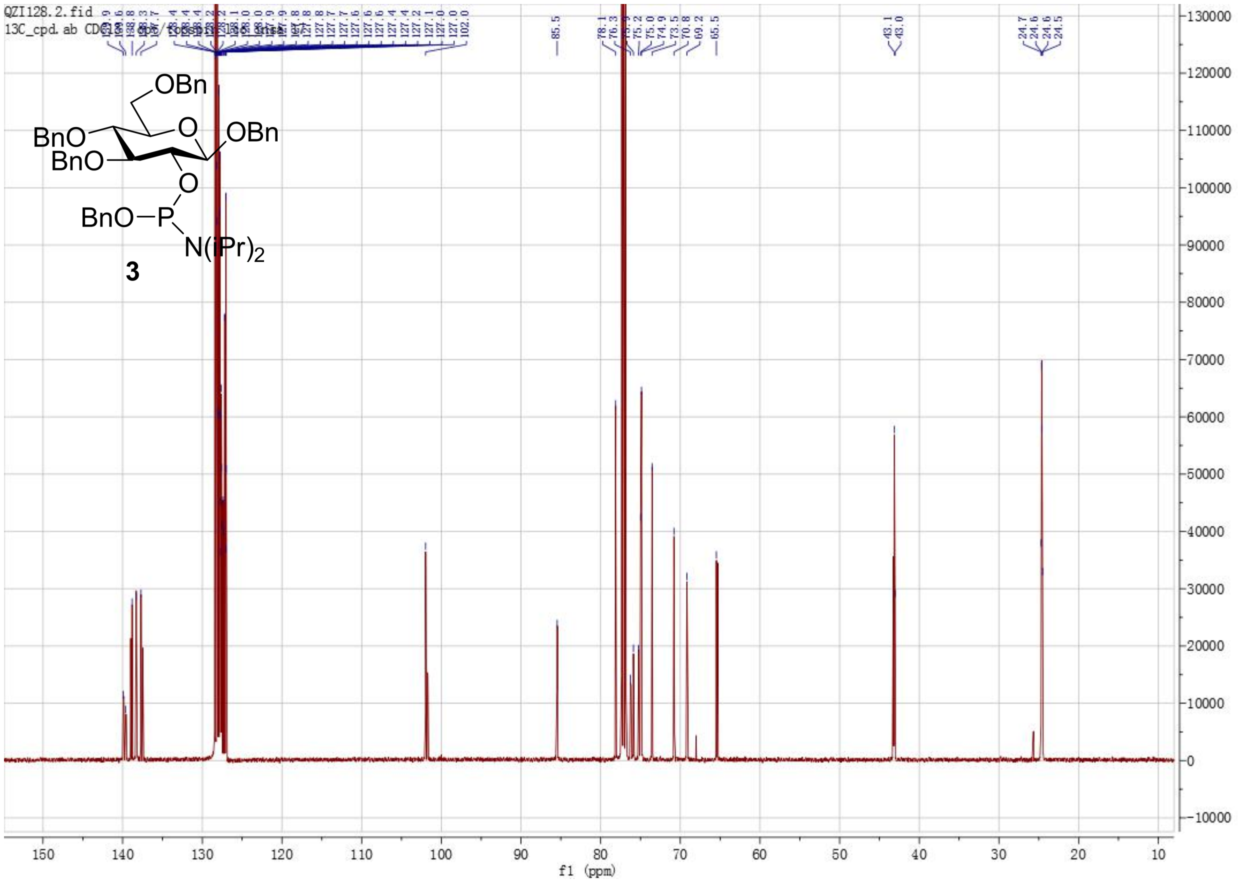
QZI128.2.fid
OBn
BnO
BnO
O
OBn
O
BnO
P
N(iPr)2
3
f1 (ppm)

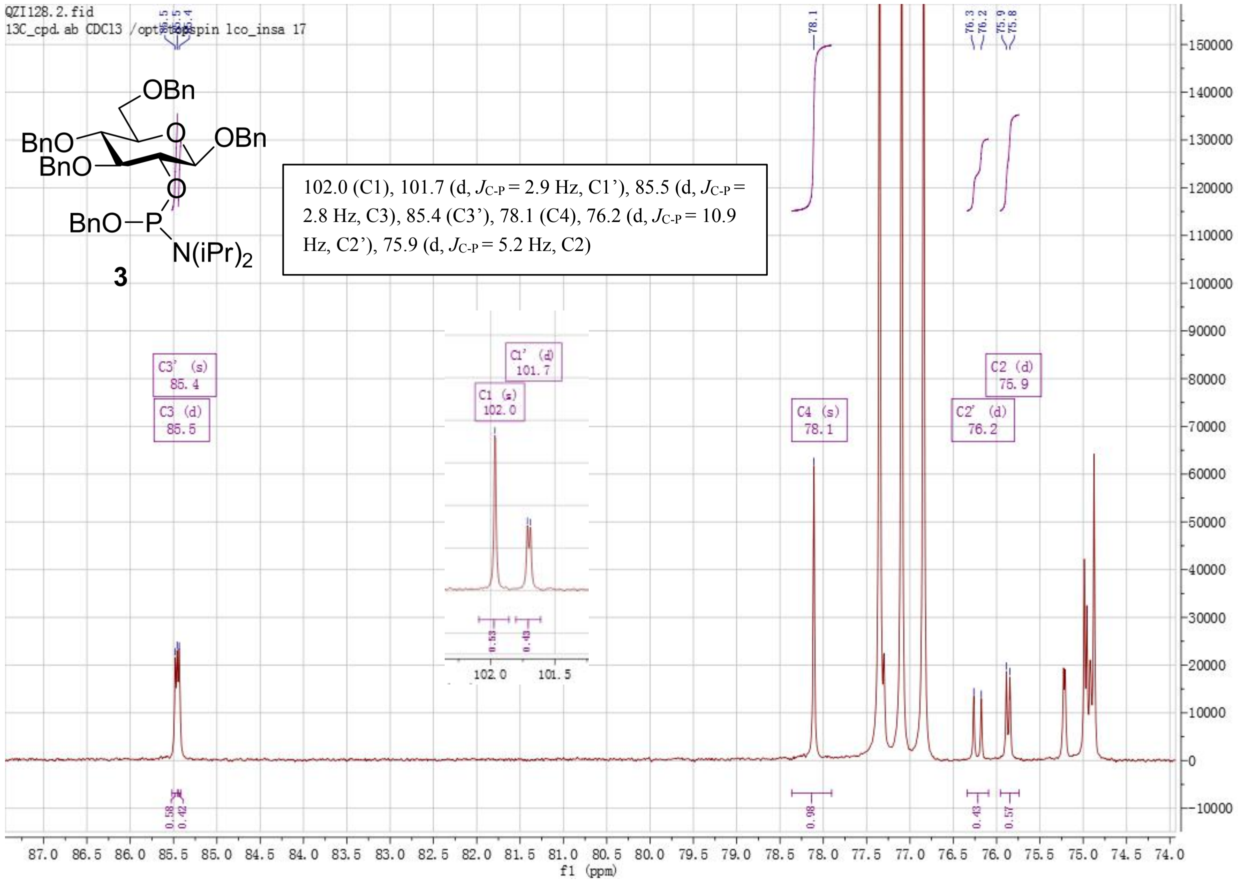
QZI128.2.fid
13C_cpd.ab CDCl3 /opt/topspin lco_insa 17
3
102.0 (C1), 101.7 (d, JC-P = 2.9 Hz, C1'), 85.5 (d, JC-P = 2.8 Hz, C3), 85.4 (C3'), 78.1 (C4), 76.2 (d, JC-P = 10.9 Hz, C2'), 75.9 (d, JC-P = 5.2 Hz, C2)
C3' (s) 85.4
C3 (d) 85.5
C1' (d) 101.7
C1 (s) 102.0
C4 (s) 78.1
C2' (d) 76.2
C2 (d) 75.9
f1 (ppm)

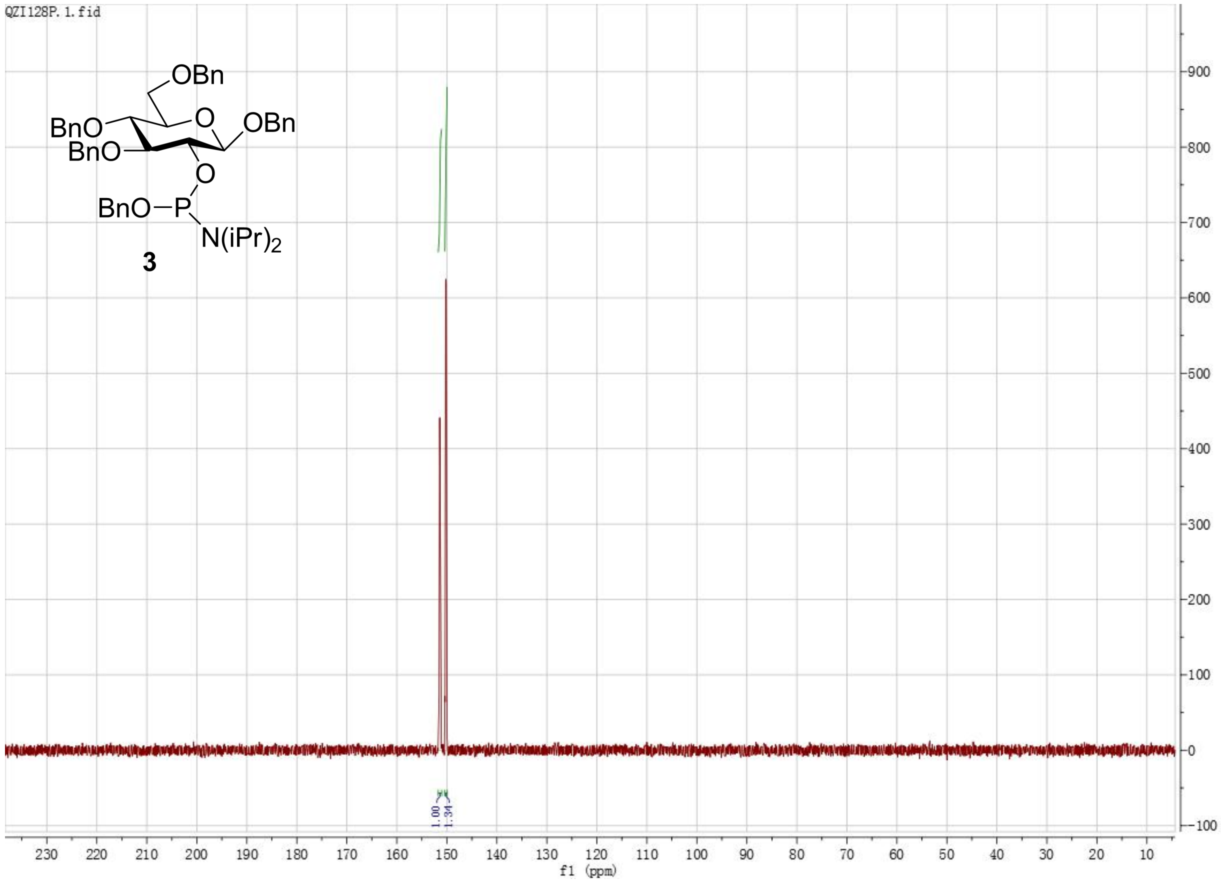
QZI128P.1.fid
OBn
BnO
O
OBn
BnO
O
BnO
P
N(iPr)2
3
f1 (ppm)

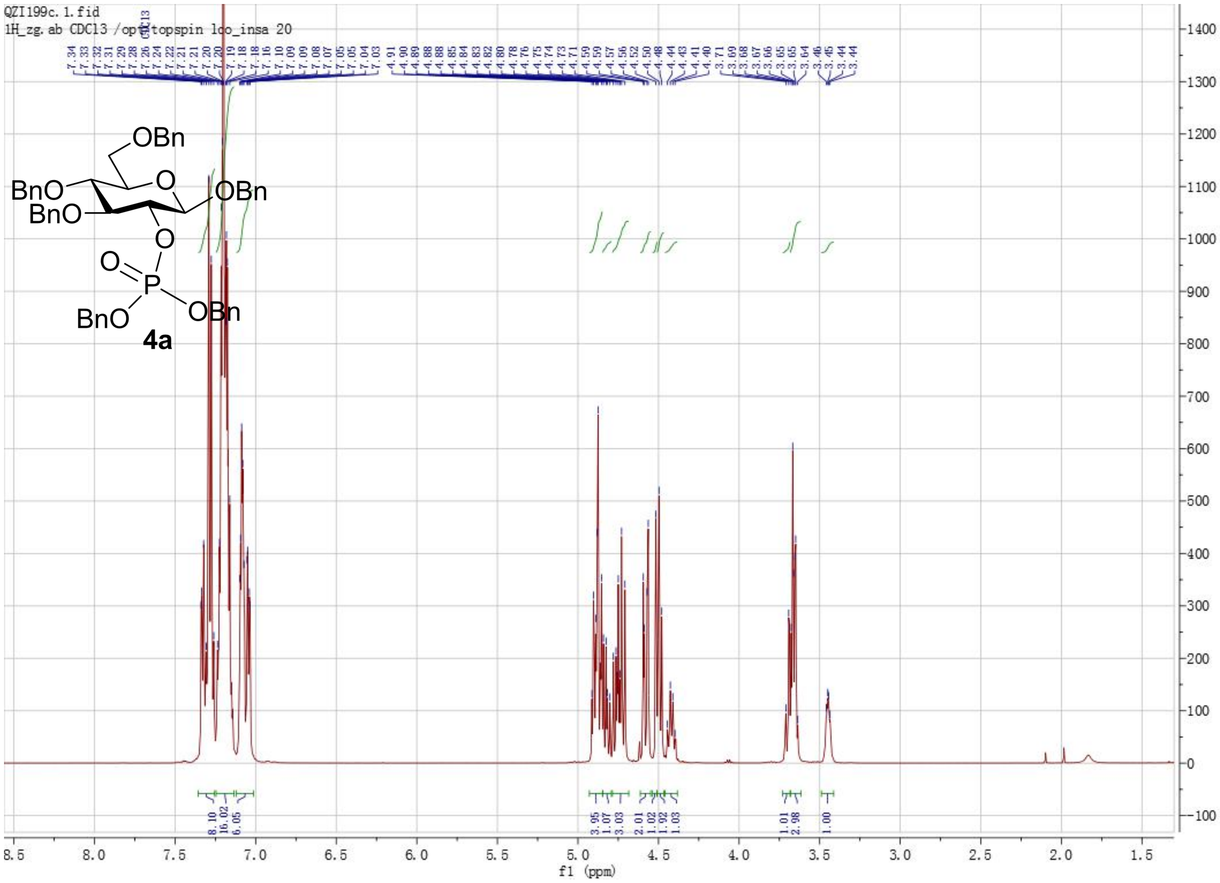
QZI199c.1.fid
1H_zg.ab CDCl3 /opt/topspin lco_insa 20
OBn
BnO
O
OBn
BnO
O
O
P
BnO
OBn
4a
f1 (ppm)

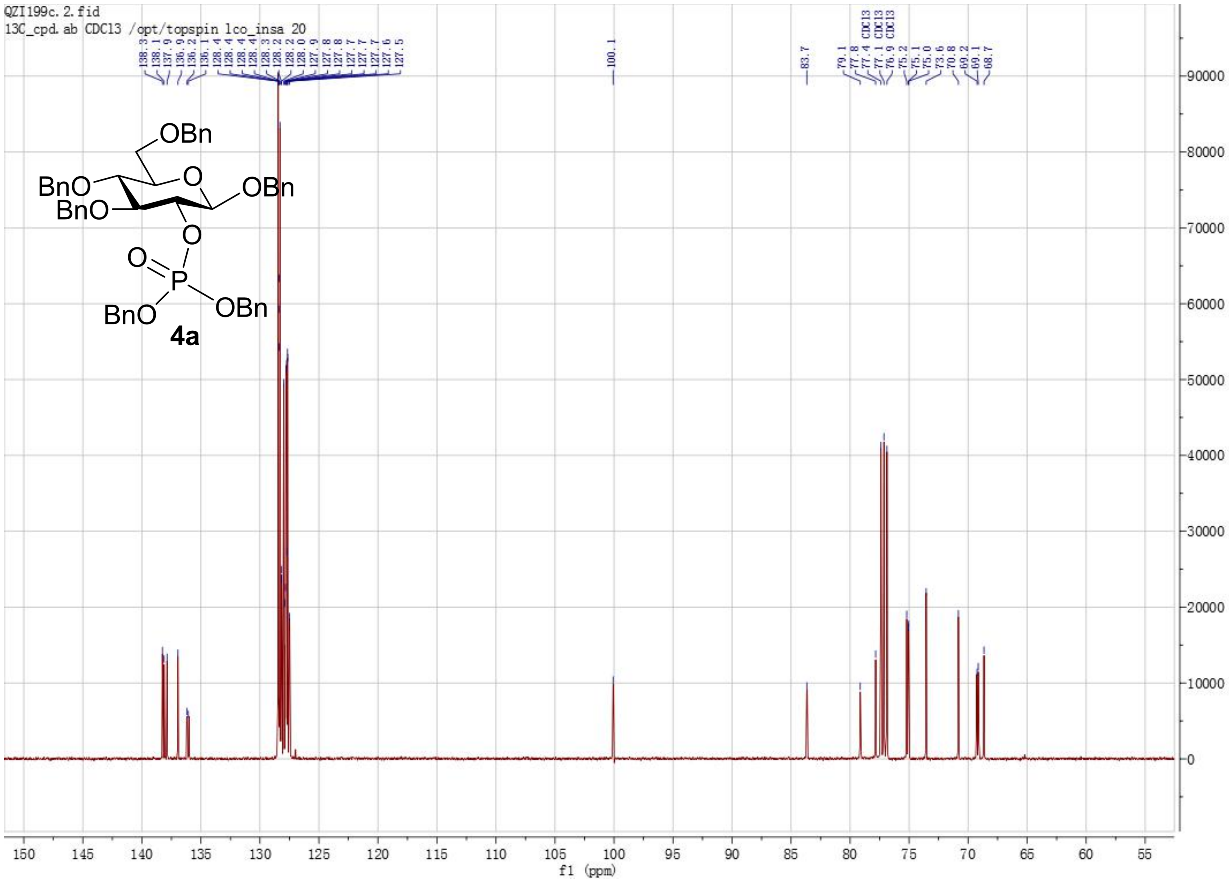
QZI199c.2.fid
13C_cpd.ab CDCl3 /opt/topspin lco_insa 20
OBn
BnO
BnO
O
OBn
O
O
P
BnO
OBn
4a
f1 (ppm)

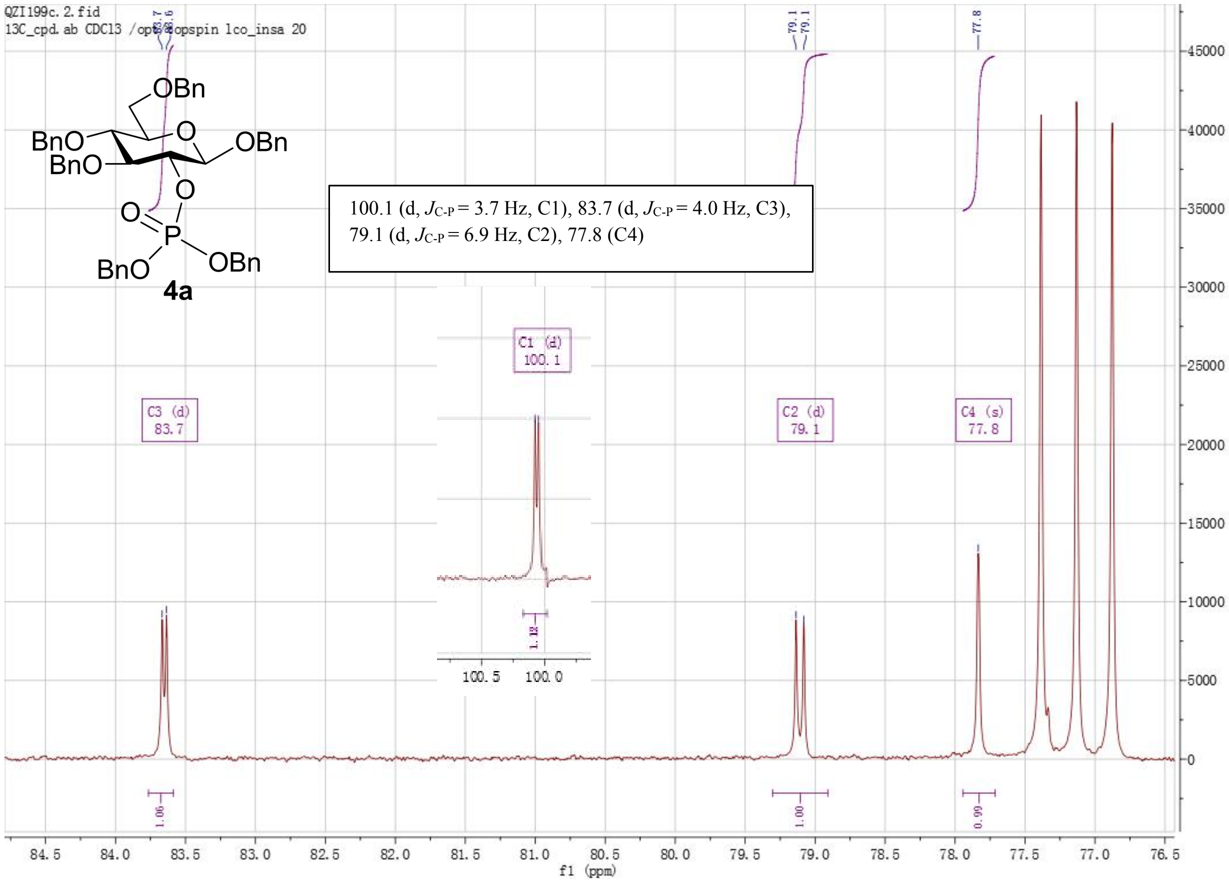
QZI199c.2.fid
13C_cpd.ab CDCl3 /opt/topspin lco_insa 20
4a
100.1 (d, J_{C-P} = 3.7 Hz, C1), 83.7 (d, J_{C-P} = 4.0 Hz, C3), 79.1 (d, J_{C-P} = 6.9 Hz, C2), 77.8 (C4)
C1 (d) 100.1
C3 (d) 83.7
C2 (d) 79.1
C4 (s) 77.8
f1 (ppm)

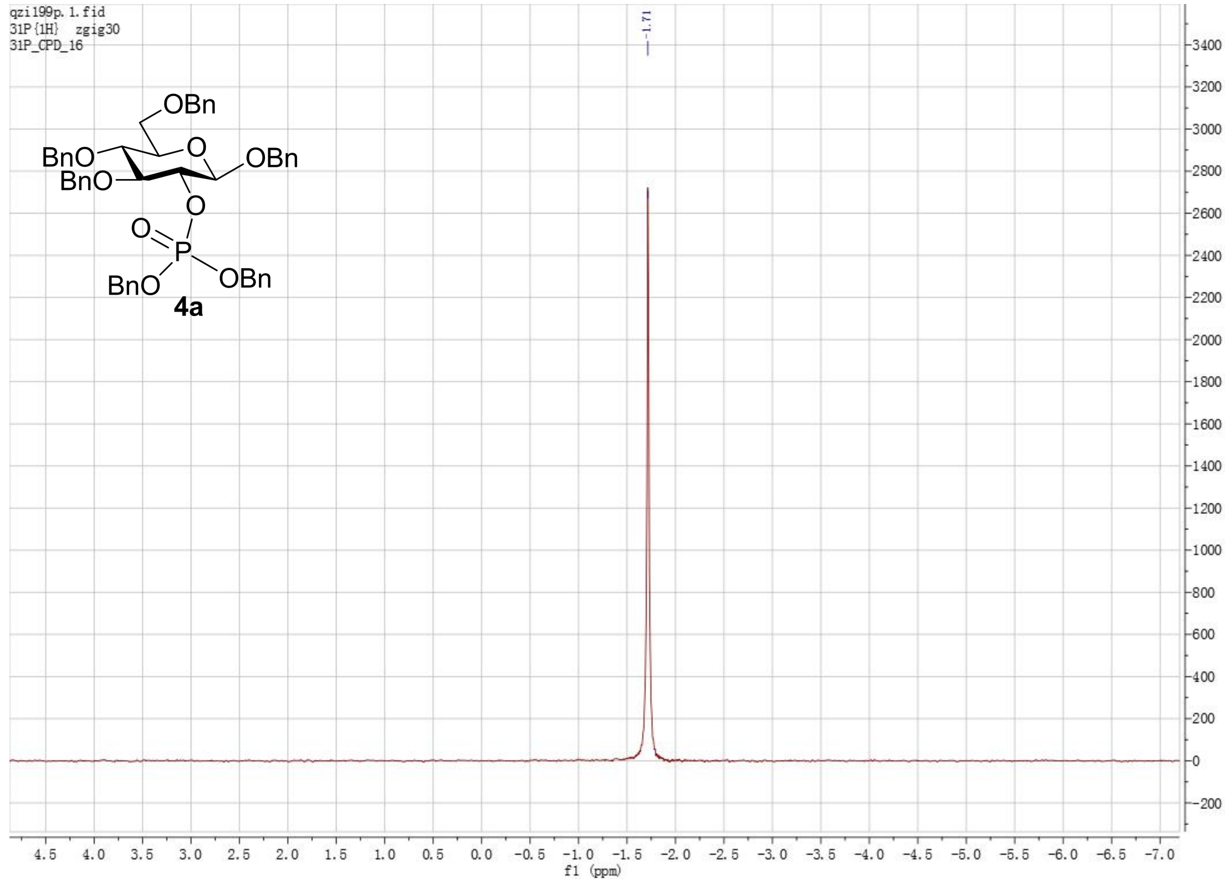

qzi199p.1.fid
31P{1H} zgig30
31P_CPD_16
-1.71
OBn
BnO
BnO
O
OBn
O
O
P
BnO
OBn
4a
f1 (ppm)

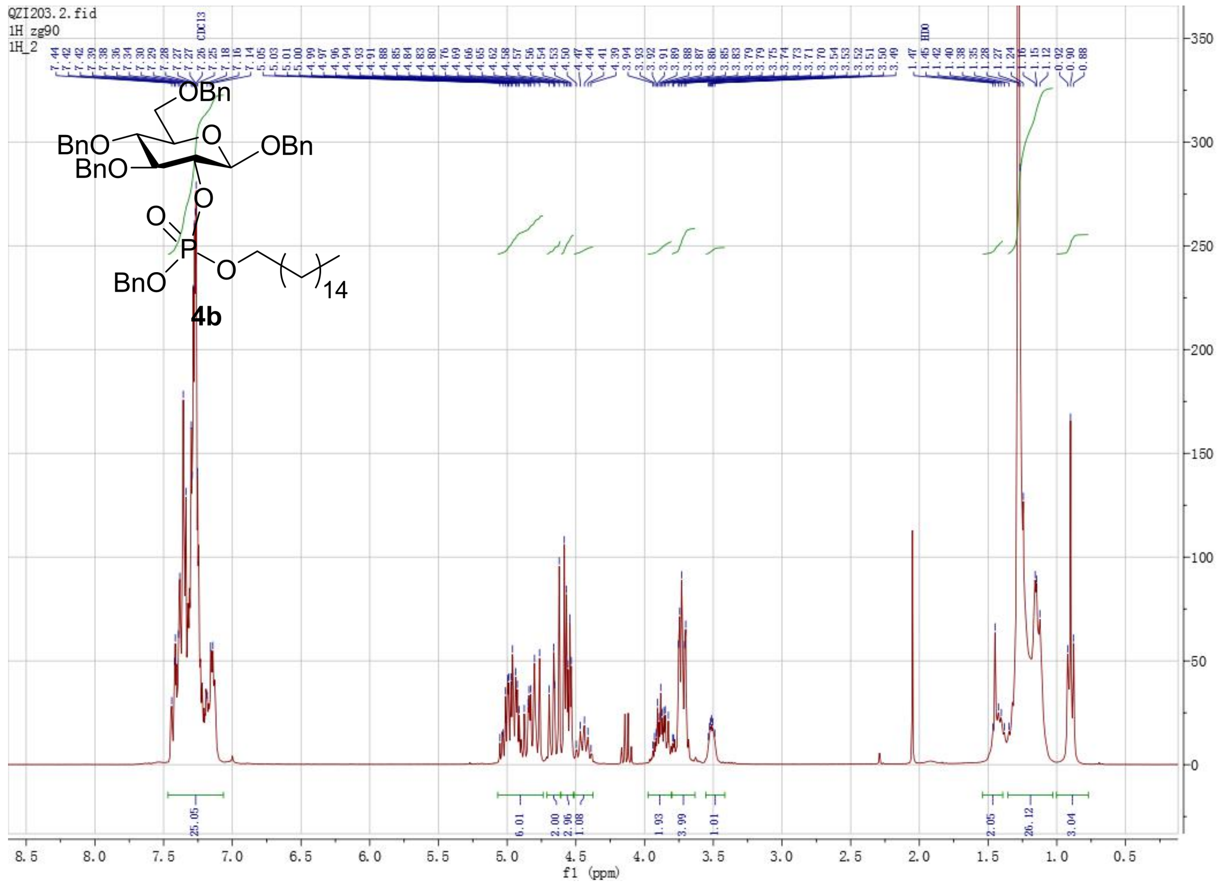

QZI203.2.fid
1H zg90
1H_2
CDCl3
HDO
OBn
BnO
BnO
O
OBn
O
O
P
BnO
O
14
4b
f1 (ppm)

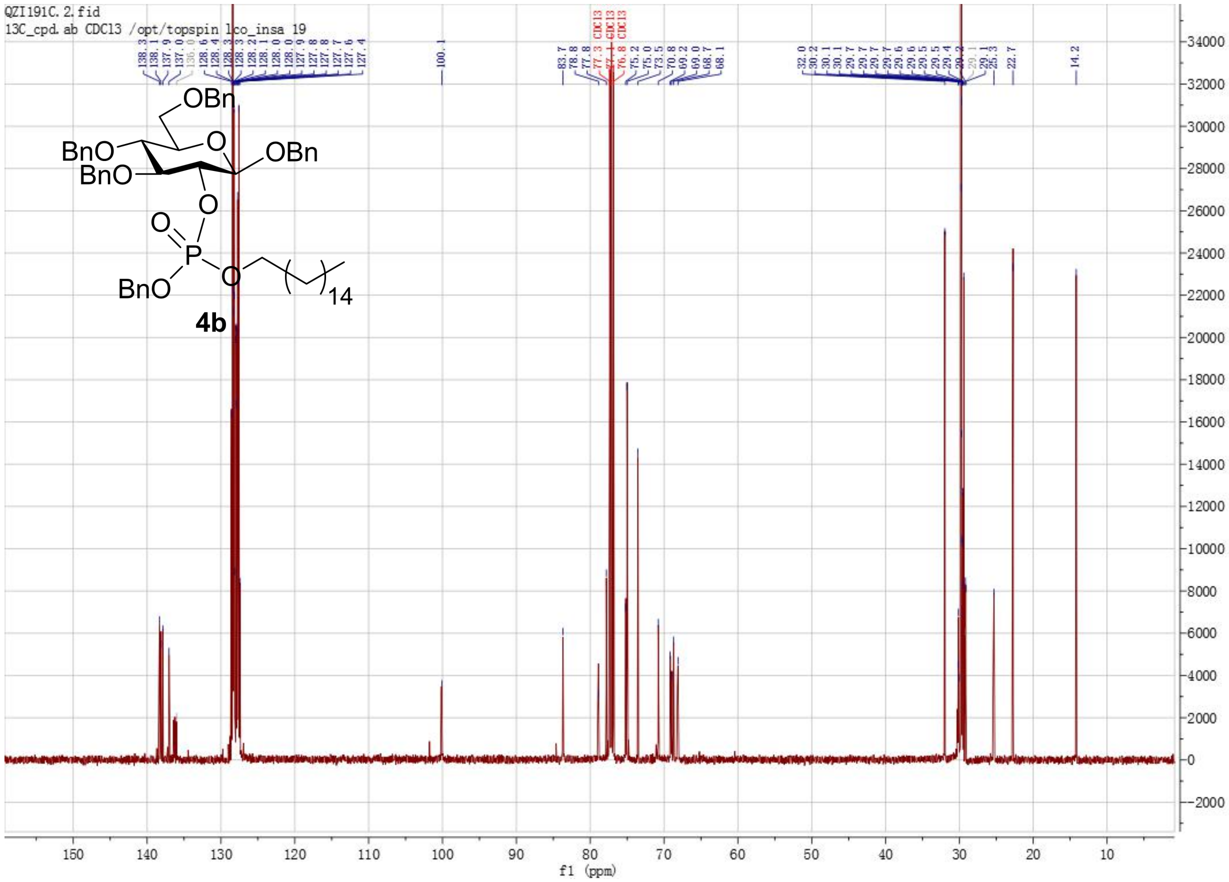
QZI191C.2.fid
13C_cpd.ab CDCl3 /opt/topspin lco_insa 19
OBn
BnO
BnO
OBn
O
P
BnO
O
14
4b
f1 (ppm)

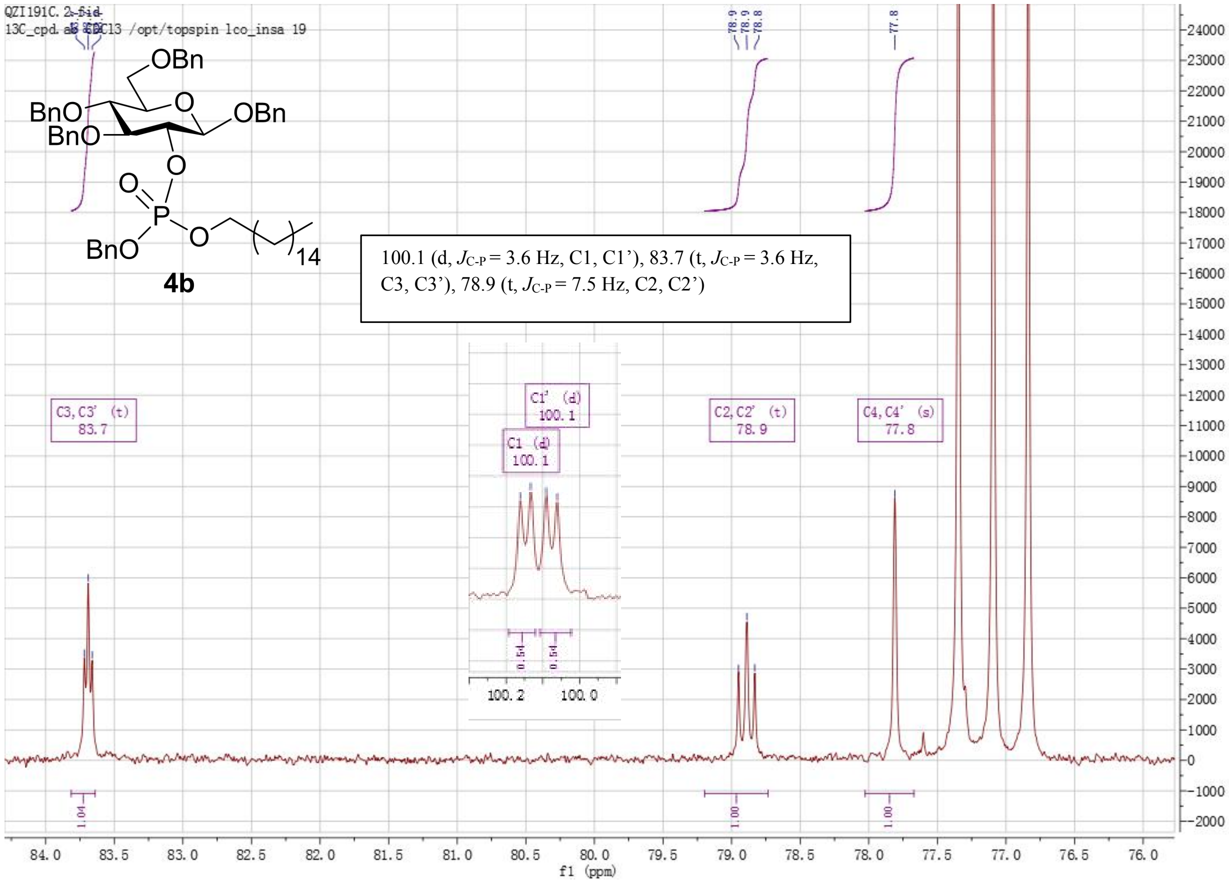
QZI191C.2.fid
13C_cpd.ab CDCl3 /opt/topspin lco_insa 19
4b
100.1 (d, J_{C-P} = 3.6 Hz, C1, C1'), 83.7 (t, J_{C-P} = 3.6 Hz, C3, C3'), 78.9 (t, J_{C-P} = 7.5 Hz, C2, C2')
C3, C3' (t) 83.7
C1' (d) 100.1
C1 (d) 100.1
C2, C2' (t) 78.9
C4, C4' (s) 77.8
f1 (ppm)

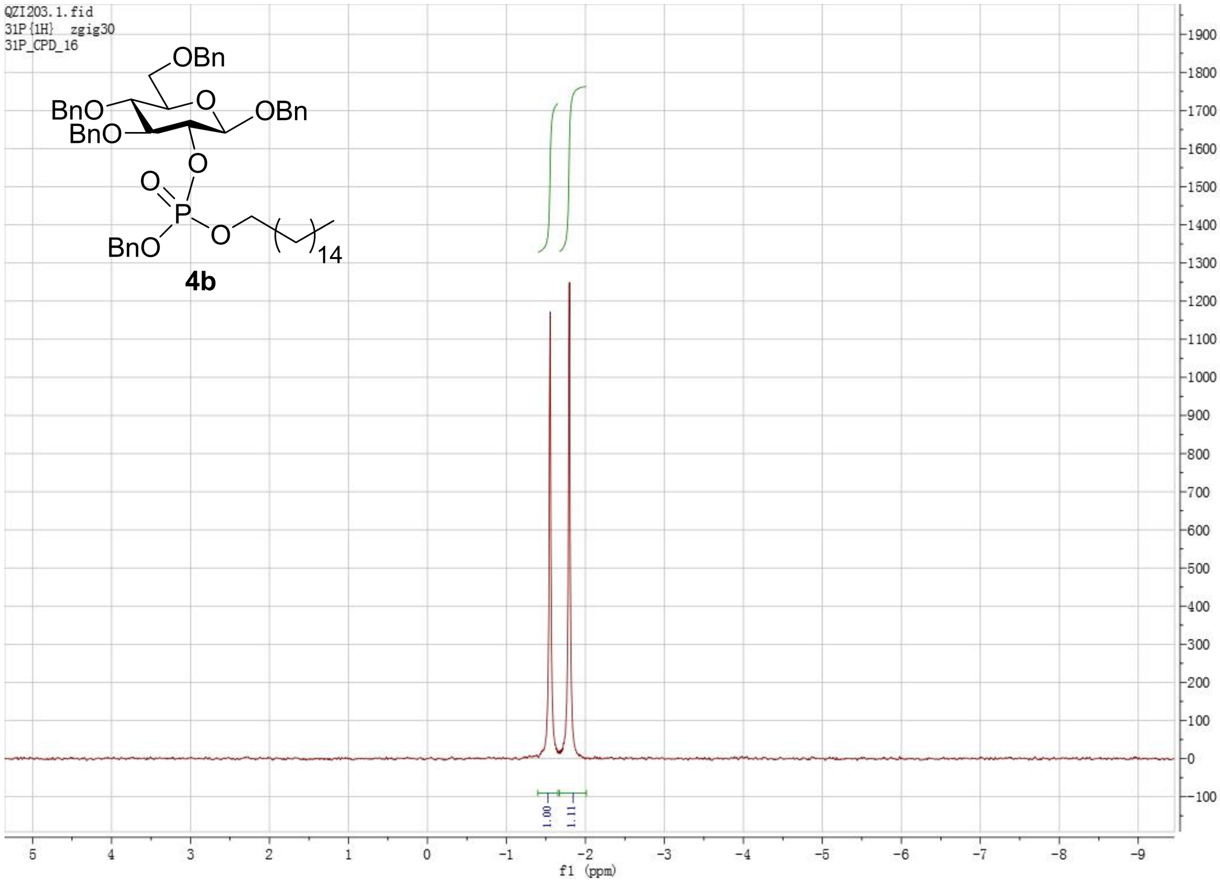
QZI203.1.fid
31P{1H} zgig30
31P_CPD_16
OBn
BnO
BnO
OBn
BnO
4b
f1 (ppm)

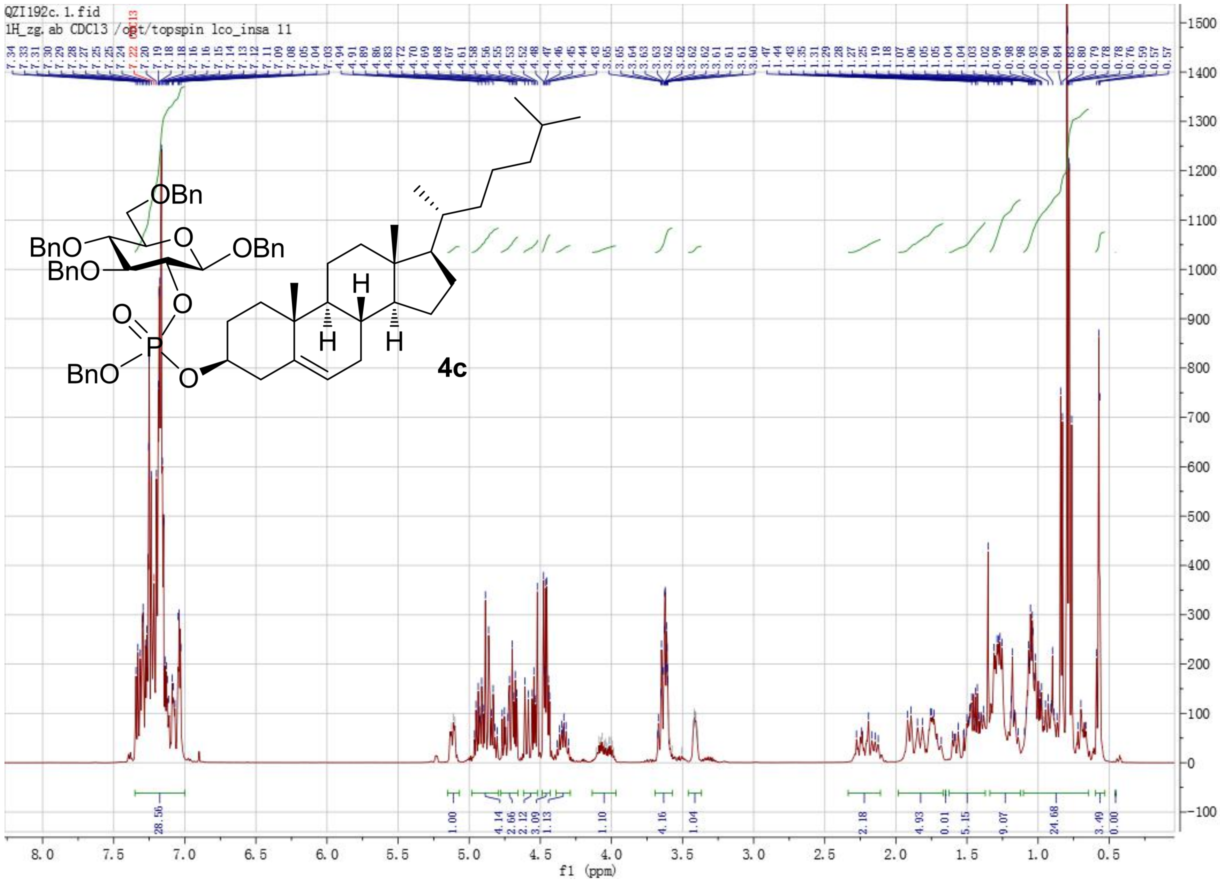
QZI192c.1.fid
1H_zg.ab CDCl3 /opt/topspin lco_insa 11
OBn
BnO
BnO
OBn
BnO
H
4c
f1 (ppm)

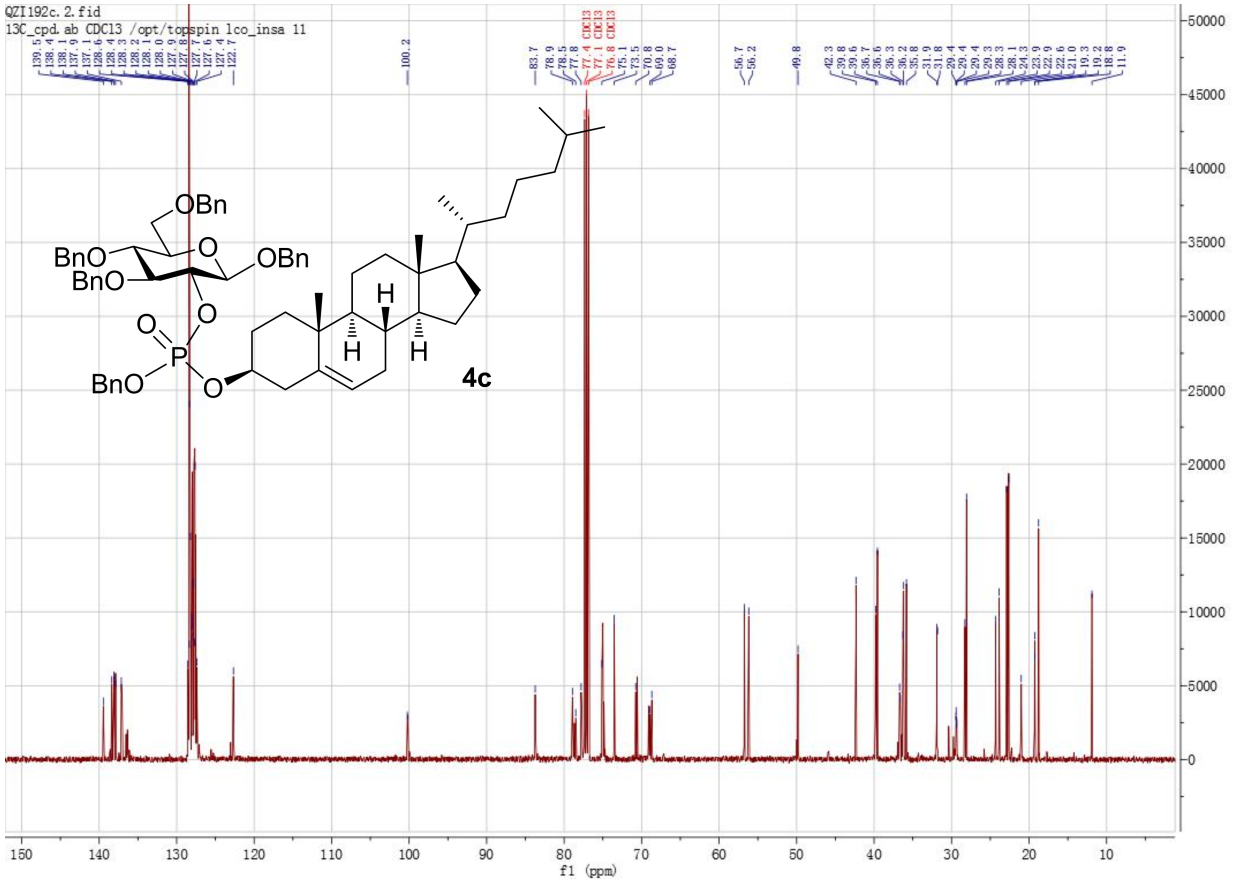
QZI192c.2.fid
13C_cpd.ab CDCl3 /opt/topspin lco_insa 11
OBn
BnO
BnO
O
OBn
O
O
P
BnO
O
H
H
H
4c
f1 (ppm)

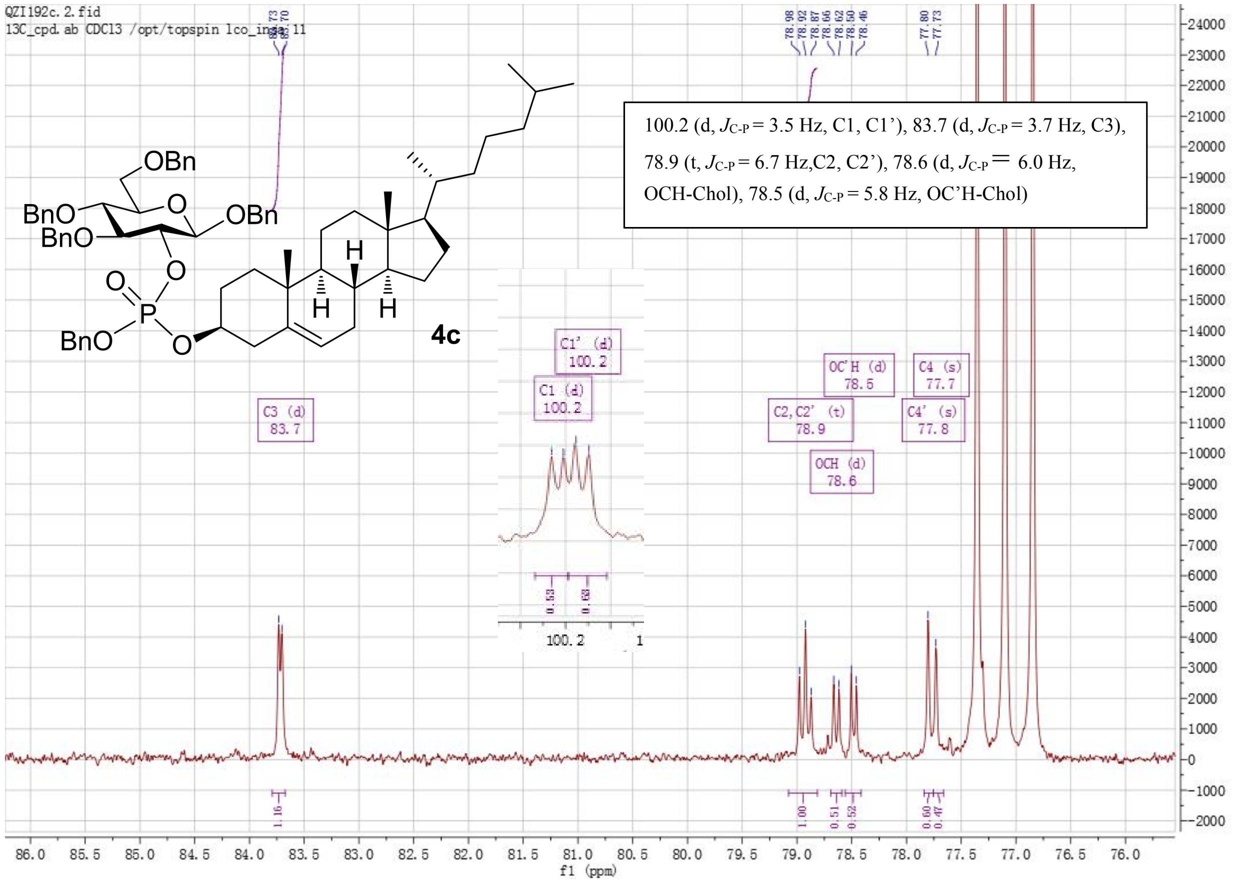
QZI192c.2.fid
13C_cpd.ab CDCl3 /opt/topspin lco_insa 11
100.2 (d, J_{C-P} = 3.5 Hz, C1, C1'), 83.7 (d, J_{C-P} = 3.7 Hz, C3), 78.9 (t, J_{C-P} = 6.7 Hz,C2, C2'), 78.6 (d, J_{C-P} = 6.0 Hz, OCH-Chol), 78.5 (d, J_{C-P} = 5.8 Hz, OC'H-Chol)
4c
C1' (d) 100.2
C1 (d) 100.2
C3 (d) 83.7
OC'H (d) 78.5
C4 (s) 77.7
C2, C2' (t) 78.9
C4' (s) 77.8
OCH (d) 78.6
f1 (ppm)

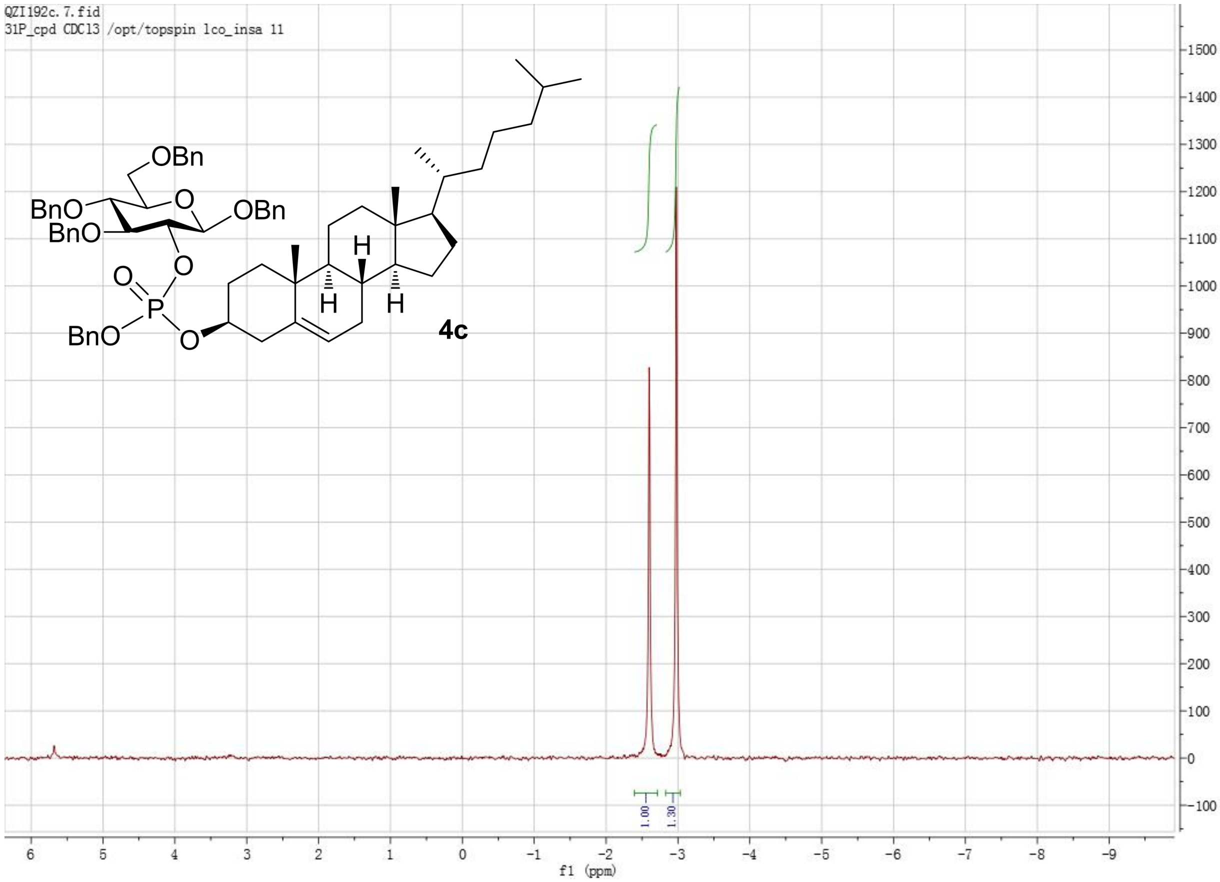
QZI192c.7.fid
31P_cpd CDCl3 /opt/topspin lco_insa 11
OBn
BnO
BnO
OBn
O
P
BnO
H
4c
f1 (ppm)

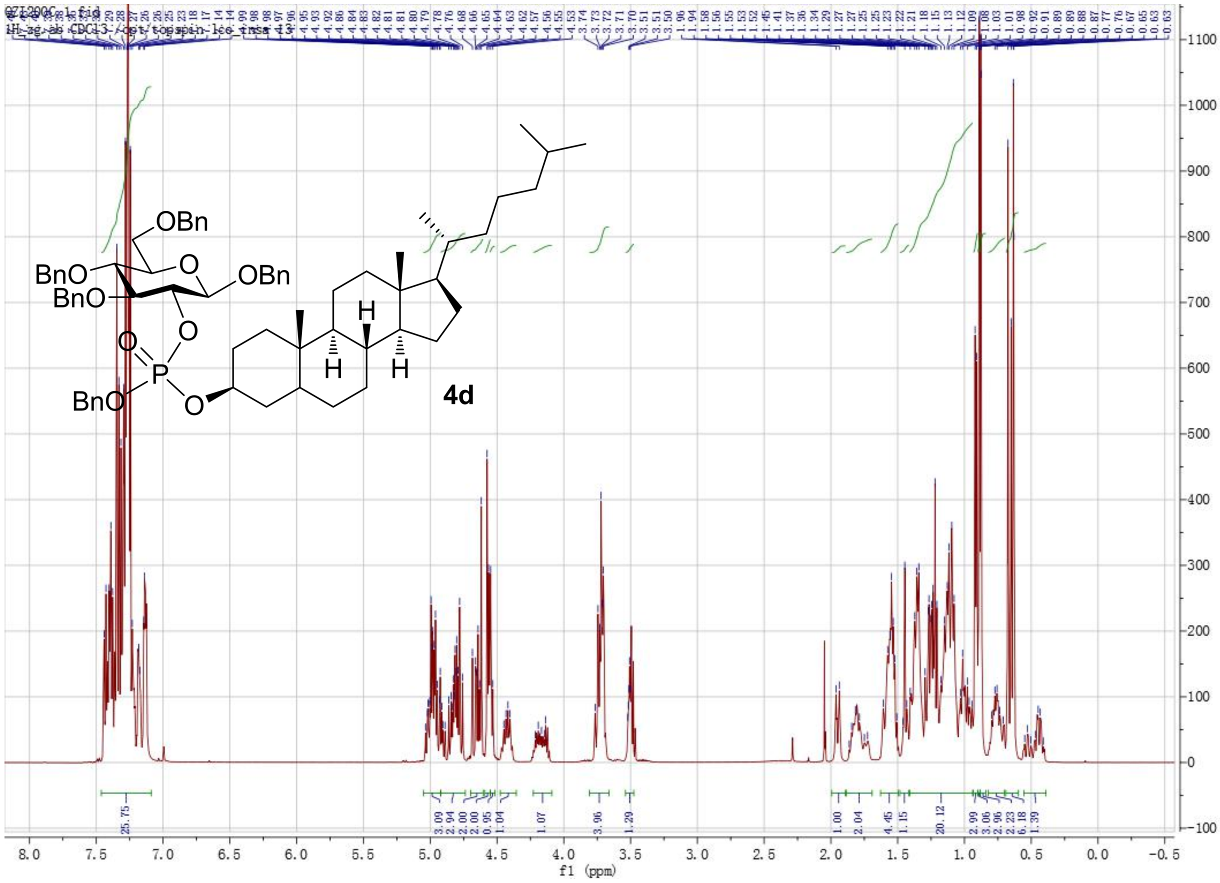
OBn
BnO
BnO
OBn
O
P
BnO
H
4d
f1 (ppm)

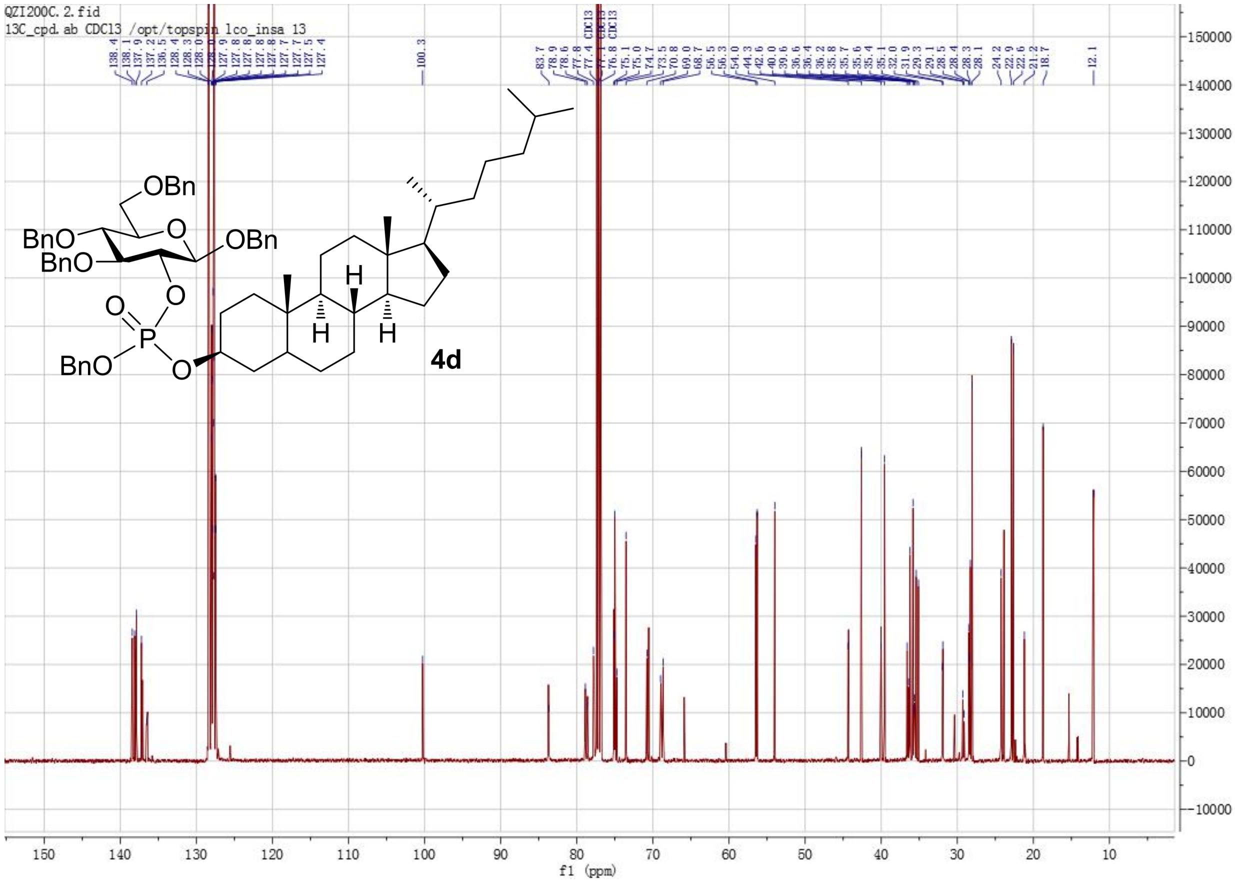
QZI200C.2.fid
13C_cpd.ab CDCl3 /opt/topspin lco_insa 13
4d
f1 (ppm)

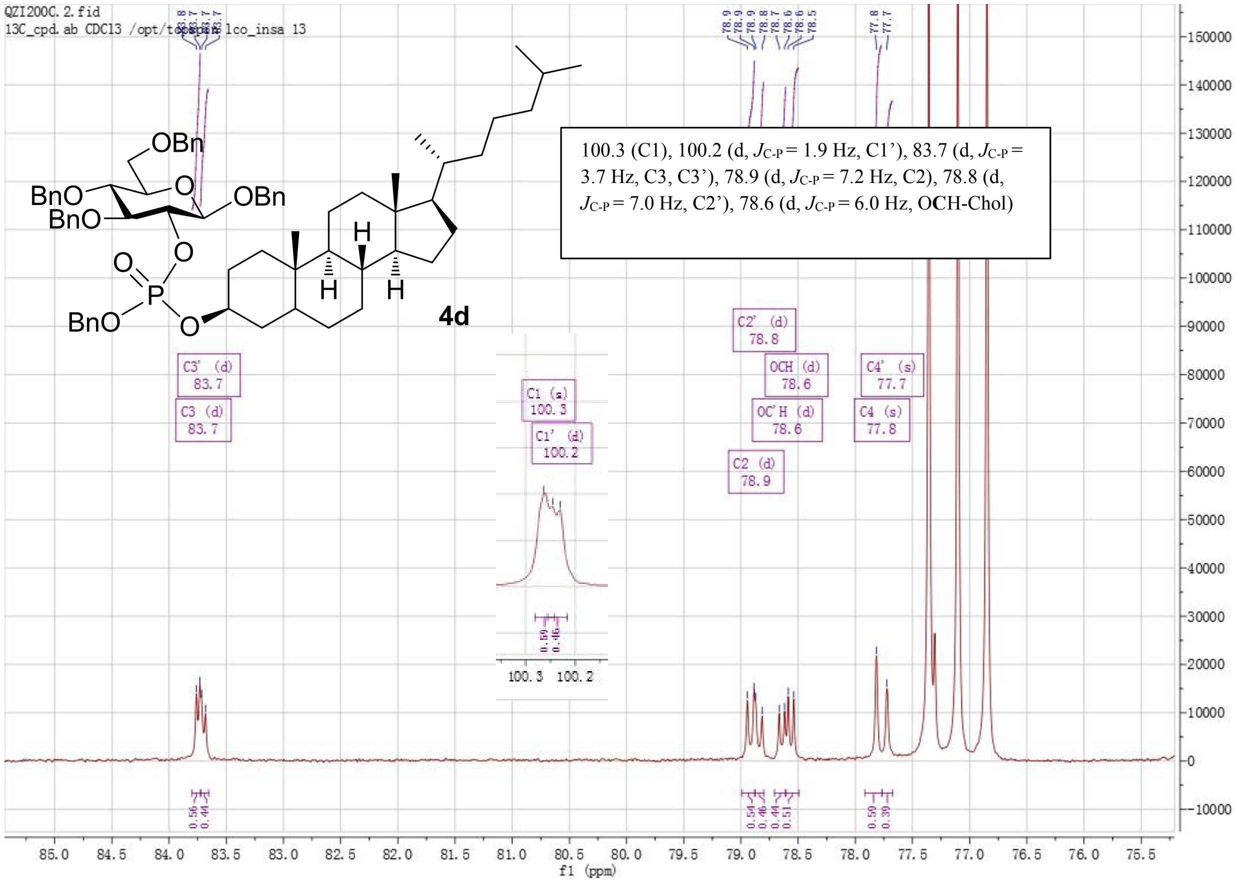
QZI200C.2.fid
13C_cpd.ab CDCl3 /opt/topspin lco_insa 13
100.3 (C1), 100.2 (d, $J_{C\text{-}P}$ = 1.9 Hz, C1'), 83.7 (d, $J_{C\text{-}P}$ = 3.7 Hz, C3, C3'), 78.9 (d, $J_{C\text{-}P}$ = 7.2 Hz, C2), 78.8 (d, $J_{C\text{-}P}$ = 7.0 Hz, C2'), 78.6 (d, $J_{C\text{-}P}$ = 6.0 Hz, OCH-Chol)
4d
C3' (d) 83.7
C3 (d) 83.7
C1 (s) 100.3
C1' (d) 100.2
C2' (d) 78.8
OCH (d) 78.6
OC'H (d) 78.6
C2 (d) 78.9
C4' (s) 77.7
C4 (s) 77.8
f1 (ppm)

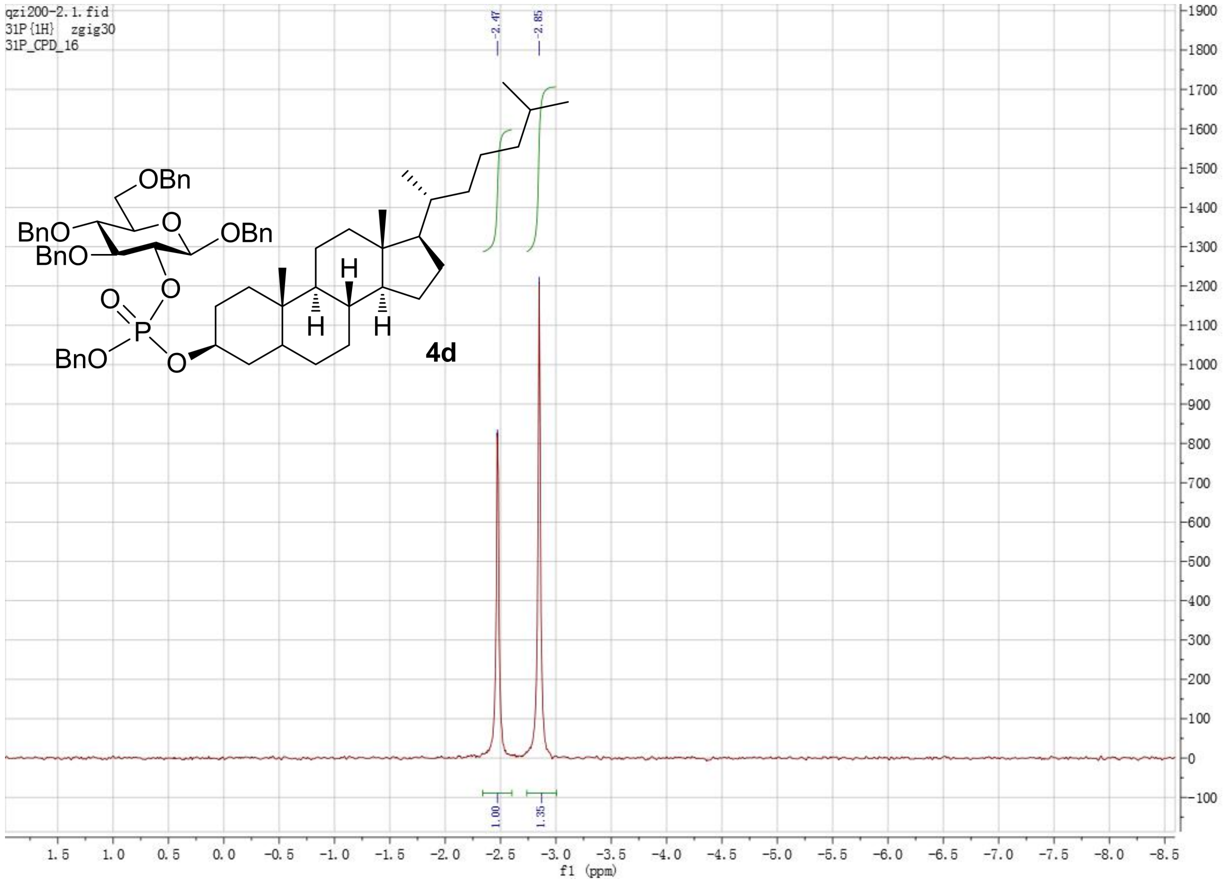
qzi200-2.1.fid
31P{1H} zgig30
31P_CPD_16
-2.47
-2.85
BnO
BnO
OBn
OBn
O
O
P
O
BnO
O
H
H
H
4d
1.00
1.35
f1 (ppm)

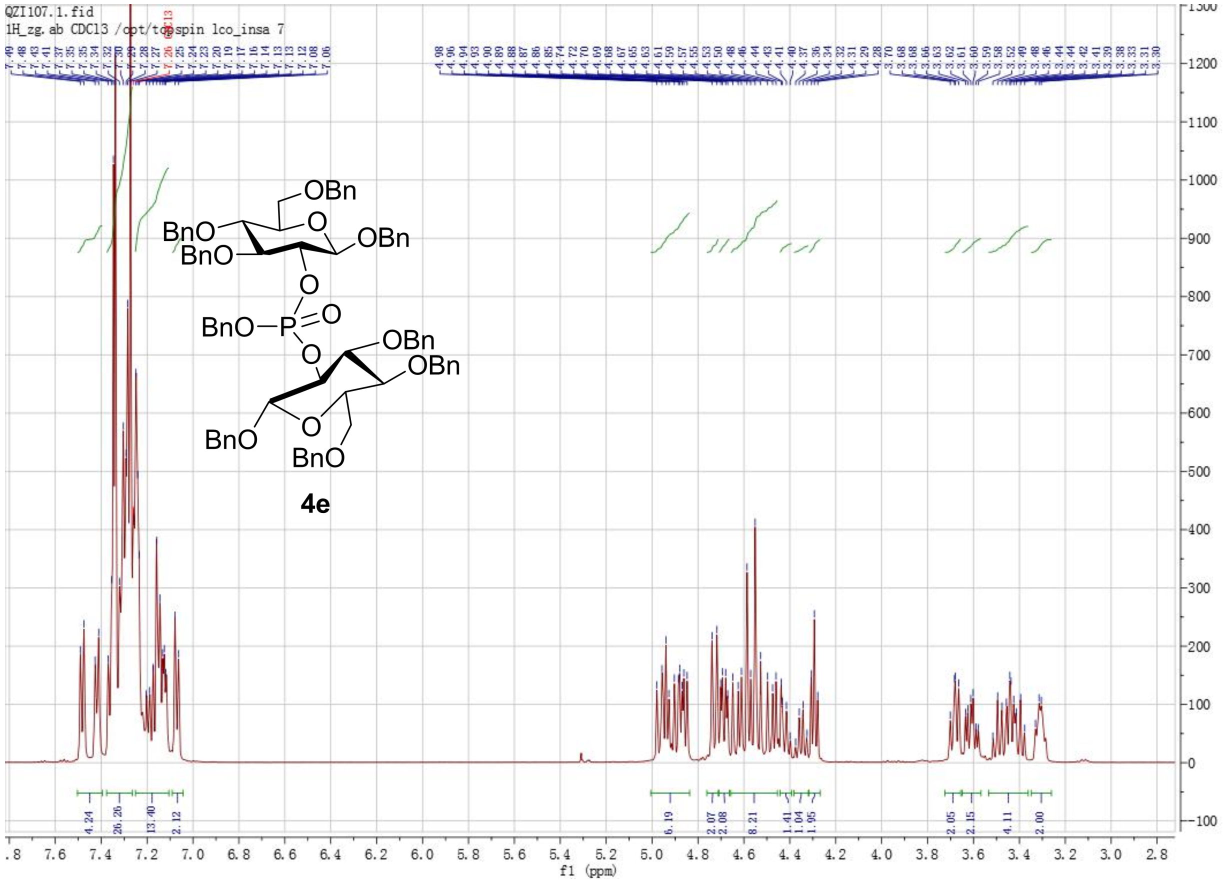
QZI107.1.fid
1H_zg.ab CDCl3 /opt/topspin lco_insa 7
OBn
BnO
BnO
OBn
O
BnO
P
O
OBn
OBn
BnO
BnO
4e
4.24
26.26
13.40
2.12
6.19
2.07
2.08
8.21
1.41
1.04
1.95
2.05
2.15
4.11
2.00
f1 (ppm)

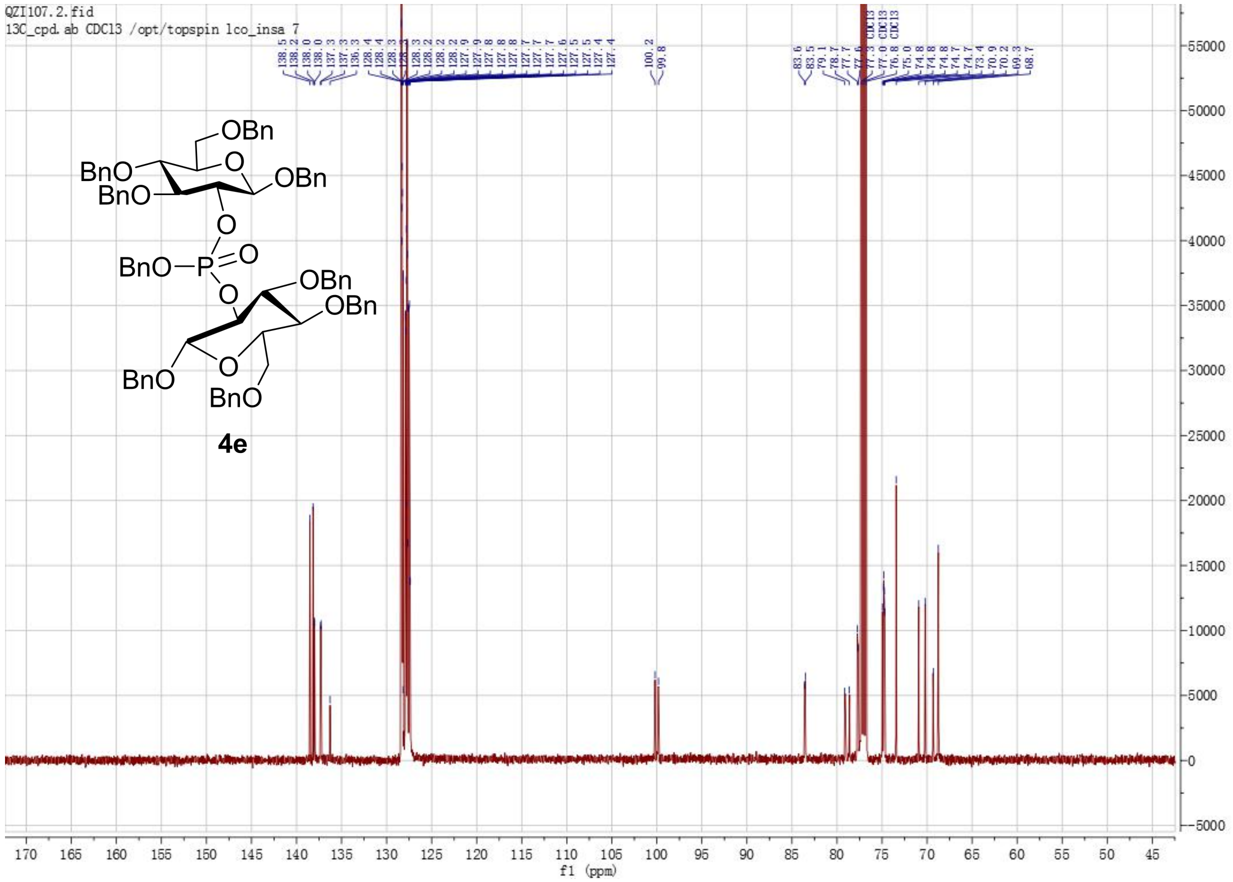
QZI107.2.fid
13C_cpd.ab CDCl3 /opt/topspin lco_insa 7
4e
f1 (ppm)

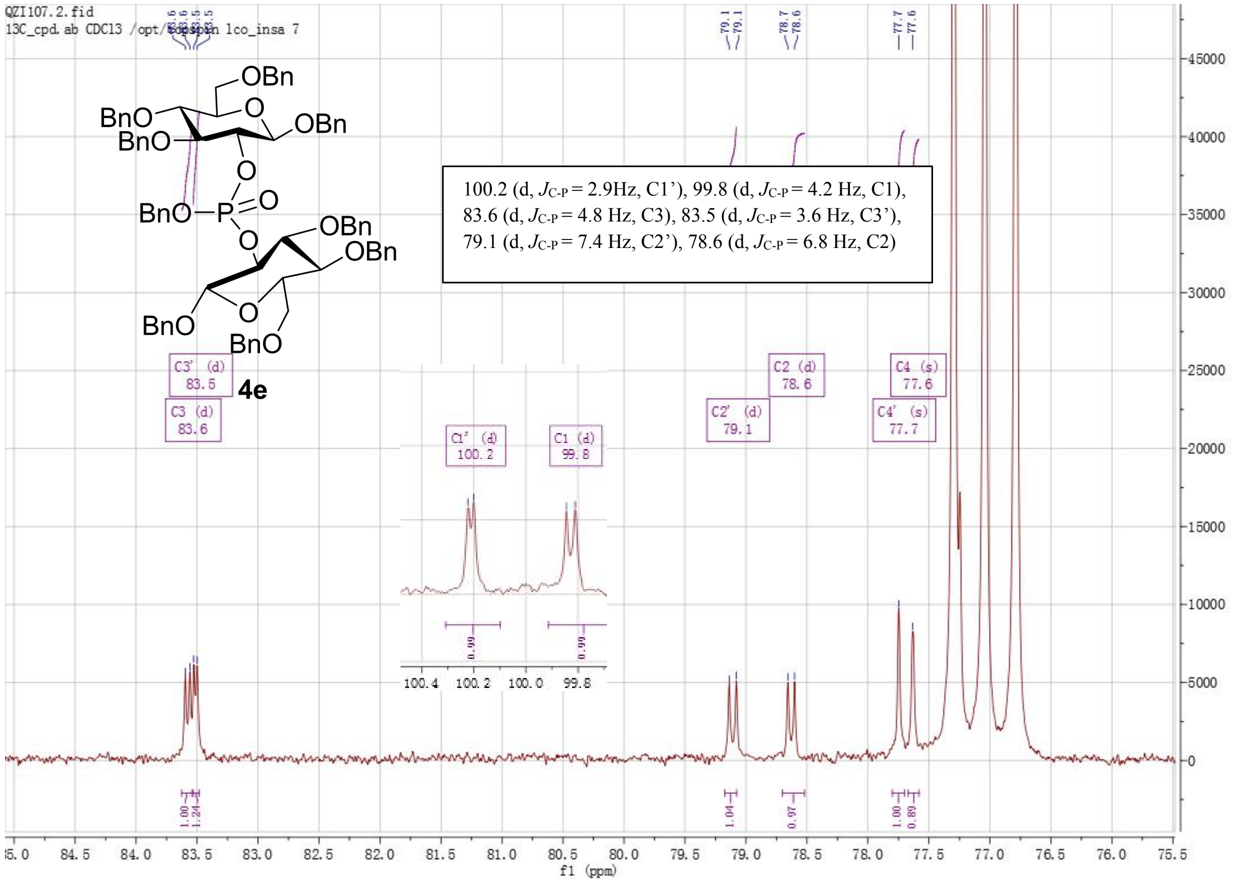
QZI107.2.fid
13C_cpd.ab CDCl3 /opt/topspin lco_insa 7
100.2 (d, J_{C-P} = 2.9Hz, C1'), 99.8 (d, J_{C-P} = 4.2 Hz, C1), 83.6 (d, J_{C-P} = 4.8 Hz, C3), 83.5 (d, J_{C-P} = 3.6 Hz, C3'), 79.1 (d, J_{C-P} = 7.4 Hz, C2'), 78.6 (d, J_{C-P} = 6.8 Hz, C2)
4e
C3' (d) 83.5
C3 (d) 83.6
C1' (d) 100.2
C1 (d) 99.8
C2' (d) 79.1
C2 (d) 78.6
C4 (s) 77.6
C4' (s) 77.7
f1 (ppm)

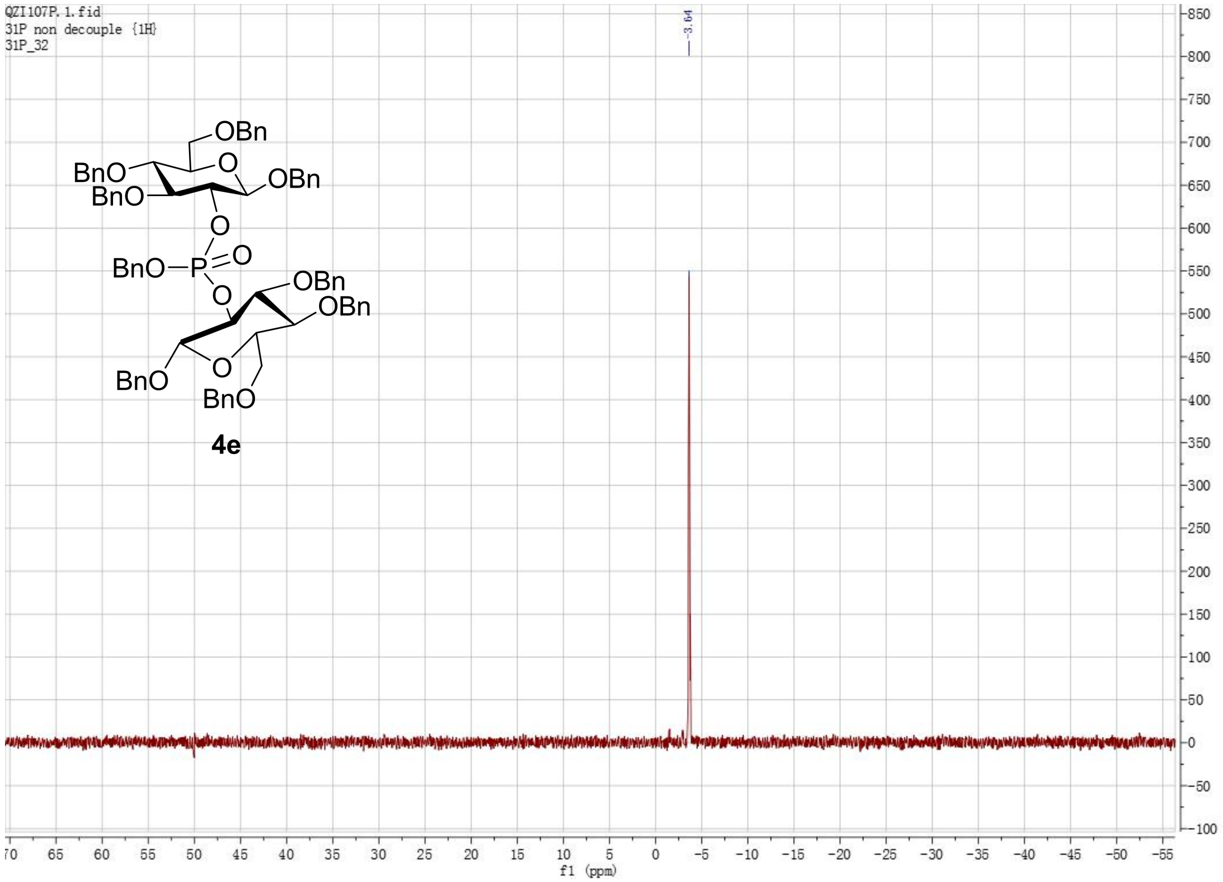
QZI107P.1.fid
31P non decouple {1H}
31P_32
OBn
BnO
O
OBn
BnO
O
BnO
P
O
O
OBn
OBn
BnO
O
BnO
—3.64
f1 (ppm)
